# Clinical factors for predicting cardiovascular risk, need for renal replacement therapy, and mortality in patients with non–dialysis-dependent stage 3–5 chronic kidney disease from the Salford Kidney Study

**DOI:** 10.1007/s40620-023-01626-8

**Published:** 2023-06-08

**Authors:** Ana Filipa Alexandre, Matthias Stoelzel, Amit Kiran, Alberto Garcia-Hernandez, Antonia Morga, Philip A. Kalra

**Affiliations:** 1 Astellas Pharma Europe B.V., Leiden, The Netherlands; 2 Astellas Pharma Europe Limited, Addlestone, UK; 3 Biostatistics Consultant, Madrid, Spain; 4 Salford Royal NHS Foundation Trust, Salford, UK; 5 Pricing and Market Access, Santen Pharmaceutical, Alpha Tower, De Entree 11-97, 1101 Amsterdam, The Netherlands

**Keywords:** Cardiovascular risk factors, Chronic kidney disease, Cohort study, Non–dialysis-dependent, Mortality

## Abstract

**Background:**

Established cardiovascular risk assessment tools lack chronic kidney disease–specific clinical factors and may underestimate cardiovascular risk in non–dialysis-dependent chronic kidney disease (CKD) patients.

**Methods:**

A retrospective analysis of a cohort of patients with stage 3–5 non–dialysis-dependent chronic kidney disease in the Salford Kidney Study (UK, 2002–2016) was performed. Multivariable Cox regression models with backward selection and repeated measures joint models were used to evaluate clinical risk factors associated with cardiovascular events (individual and composite cardiovascular major adverse cardiovascular events), mortality (all-cause and cardiovascular-specific), and need for renal replacement therapy. Models were established using 70% of the cohort and validated on the remaining 30%. Hazard ratios ([95% CIs]) were reported.

**Results:**

Among 2192 patients, mean follow-up was 5.6 years. Cardiovascular major adverse cardiovascular events occurred in 422 (19.3%) patients; predictors included prior history of diabetes (1.39 [1.13–1.71]; *P* = 0.002) and serum albumin reduction of 5 g/L (1.20 [1.05–1.36]; *P* = 0.006). All-cause mortality occurred in 740 (33.4%) patients, median time to death was 3.8 years; predictors included reduction of estimated glomerular filtration of 5 mL/min/1.73 m^2^ (1.05 [1.01–1.08]; *P* = 0.011) and increase of phosphate of 0.1 mmol/L (1.04 [1.01–1.08]; *P* = 0.021), whereas a 10 g/L hemoglobin increase was protective (0.90 [0.85–0.95]; *P* < 0.001). In 394 (18.0%) patients who received renal replacement therapy, median time to event was 2.3 years; predictors included halving of estimated glomerular filtration rate (3.40 [2.65–4.35]; *P* < 0.001) and antihypertensive use (1.23 [1.12–1.34]; *P* < 0.001). Increasing age, albumin reduction, and prior history of diabetes or cardiovascular disease were risk factors for all outcomes except renal replacement therapy.

**Conclusions:**

Several chronic kidney disease–specific cardiovascular risk factors were associated with increased mortality and cardiovascular event risk in patients with non–dialysis-dependent chronic kidney disease.

**Graphical abstract:**

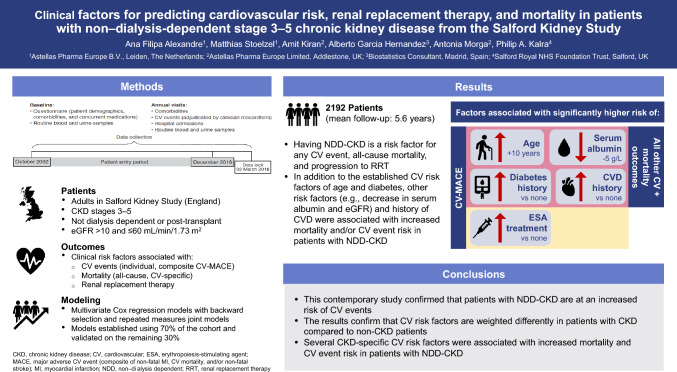

**Supplementary Information:**

The online version contains supplementary material available at 10.1007/s40620-023-01626-8.

## Introduction

Chronic kidney disease (CKD) increases the risk of cardiovascular disease (CVD) [1–3]. Cardiovascular disease prevalence is up to nine times higher in patients with CKD in the United States, and cardiovascular (CV) mortality is also elevated in patients with CKD compared with the general population [4, 5]. Globally, 1.4 million CVD-related deaths (7.6% of CVD deaths) were attributable to impaired kidney function in 2017 [6].

The Framingham Risk Equation predicts CV risk using traditional CV risk factors: age, sex, blood pressure, cholesterol, diabetes, and smoking [7, 8]. However, it was not developed for patients with CKD, it underestimates CVD risk in this population, and it does not account for effects or complications of low renal function or concurrent CKD on traditional CV risk factor duration or severity [7–9]. Additionally, CV risk factors in the Framingham Risk Equation appear to carry different weights in patients with CKD (e.g., diabetes influences CV events more than high cholesterol or hypertension) [8]. Non-traditional CV risk factors (e.g., anemia, albuminuria, mineral and bone metabolic abnormalities) also may contribute to increased CVD risk in patients with CKD [5, 10, 11].

The addition of an estimated glomerular filtration rate (eGFR) of < 60 mL/min/1.73 m^2^ to the Framingham Risk Equation may not improve its predictive ability in patients with CKD [7], highlighting the need for CKD-specific CV risk equations [8]. Understanding the influence of CV risk factors in the CKD population may support the development of such an equation. This study aimed to evaluate clinical and laboratory risk factors associated with major adverse CV events (CV-MACE), any CV event, CV mortality (CVM), all-cause mortality (ACM), and progression to renal replacement therapy (RRT) in patients with non–dialysis-dependent (NDD)-CKD.

## Methods

### Study design and data source

The Salford Kidney Study (SKS) is an ongoing (2002–present), prospective study of patients with CKD referred to renal services at Salford Royal National Health Service (NHS) Foundation Trust in northwest England [12], which serves a catchment population of approximately 1.55 million. Adults (≥ 18 years) referred to the Trust’s renal services with an eGFR < 60 mL/min/1.73 m^2^ are eligible to join. Patients are enrolled in the SKS after written informed consent is obtained.

### Study cohort

Patients in the SKS between October 2002 and December 2016 with NDD-CKD and eGFR > 10 and ≤ 60 mL/min/1.73 m^2^ recorded in the 12 months preceding their recruitment date were included. Exclusion reasons included receiving dialysis or a kidney transplant or not having recruitment date, sex, or baseline age recorded.

### Data collection

Patients were followed annually from recruitment until death, RRT initiation, or data lock (March 2, 2018) (Fig. S1).

Biochemical parameters collected included hemoglobin, hematocrit, ferritin, transferrin saturation, eGFR, creatinine, phosphate, corrected calcium, random glucose, parathyroid hormone, C-reactive protein (CRP), albumin, and urinary protein:creatinine ratio (Table S1). The MDRD (Modification of Diet in Renal Disease) equation was used to calculate eGFR [13]. Anemia treatment was initiated when clinically appropriate (Online Resource 1).

Mortality was established using date of death from the NHS Spine database and cause of death from the Office of National Statistics death certificate or Salford electronic patient record. Additional data collection details are provided in Online Resource 1.

### Study outcomes

The primary outcome was first occurrence of CV-MACE, defined as the earliest date of non-fatal myocardial infarction (MI) diagnosis, non-fatal stroke diagnosis, or death due to a CV event (assumed if CV death was indicated in the cause-of-death portion of the dataset).

Secondary outcomes were first occurrence of any CV event (MI, unstable angina, coronary revascularization therapy, congestive cardiac failure [CCF], or stroke) or ACM, first occurrence of MI, first occurrence of non-fatal stroke, CVM, ACM, and occurrence of RRT.

### Statistical analysis

Baseline demographics and clinical characteristics were summarized descriptively. The sample was bifurcated by random selection: 70% was used to build a predictive model and 30% was used for independent model validation.

Time-to-event analyses were performed for each outcome. Kaplan–Meier plots stratified by CKD stage at baseline were generated, then a proportional hazards (PH) model was fitted, and the best-fitting baseline hazard function (assessed using Bayesian information criterion) was adopted.

Univariate analyses were performed to determine whether a baseline clinical or laboratory parameter predicted a study outcome. A multivariable PH model was developed including all statistically significant parameters from the univariate analyses and clinically important variables, followed by a backward selection procedure. The associations of risk factors with outcomes were investigated in a joint model [14] that combined a repeated measures model for eGFR and the multivariable PH model for risk factors at baseline. Additional details on statistical analyses and post hoc analyses are described in Online Resource 1.

### Missing data

Missing event dates and key variables were imputed as described in Online Resource 1.

### Model validation

The model was assessed on the validation cohort for positive and negative predictive value using area under receiver operating characteristics curves (AUCs) as summary measures of the model’s accuracy. The joint model was used to predict the cumulative 3-, 5-, and 10-year risk of events in the validation cohort for patients who had an event (to obtain sensitivity) and those who did not have an event (to obtain specificity).

## Results

In total, 3132 patients were included in this analysis (Fig. S2). Most patients were male, the mean age at baseline was 64.1 years, mean eGFR was 33.2 mL/min/1.73 m^2^, 57.7% had ferritin levels > 100 µg/L, and 86.5% were not treated with an erythropoiesis-stimulating agent (ESA) (Table 1). Mean follow-up time was 4.8 years (maximum, 15.4 years).

**Table 1:** Patient characteristics at baseline

Parameter	Total population (*N* = 3132)
Sex, male, *n* (%)	1951 (62.3)
Ethnic group, *n* (%) [*n* = 3126]	
White	3002 (96.0)
Asian	80 (2.6)
Black	30 (1.0)
Other	14 (0.5)
Age, years	
Mean (SD)	64.1 (14.7)
Median (min, max)	67 (18, 94)
Weight, mean (SD), kg [*n* = 2614]	82.4 (18.8)
BMI, mean (SD), kg/m^2^ [*n* = 2571]	28.94 (6.1)
Smoking status, *n* (%)	
Active	387 (12.4)
Former	1671 (53.4)
Non-smoker	1074 (34.3)
Renal diagnosis, *n* (%)	
Diabetic nephropathy	594 (19.0)
Hypertensive nephrosclerosis	396 (12.6)
Renovascular disease	249 (8.0)
Glomerulonephritis	388 (12.4)
Polycystic kidney disease	157 (5.0)
Chronic pyelonephritis	194 (6.2)
Unknown	419 (13.4)
Others	535 (17.1)
Missing	200 (6.4)
CKD stage, *n* (%) [*n* = 2141]	
3a	369 (17.2)
3b	650 (30.4)
4	772 (36.1)
5	193 (9.0)
eGFR, mL/min/1.73 m^2^ [*n* = 2141]	
Mean (SD)	33.2 (15.6)
Median (min, max)	31 (6, 90)
Hb, mean (SD), g/L [*n* = 2862]	123.6 (17.0)
Hb < 100 g/L, n (%) [*n* = 2862]	201 (7.0)
LDL cholesterol, mean (SD), mmol/L [*n* = 2179]	2.4 (1.2)
Comorbidities, *n* (%)	
Diabetes mellitus	1037 (33.1)
CVD	1083 (34.6)
Cerebrovascular disease	255 (8.1)
PVD	432 (13.8)
LVH	29 (0.9)
Malignancy	424 (13.5)
SBP, mean (SD), mmHg [*n* = 3093]	140.3 (21.7)
DBP, mean (SD), mmHg [*n* = 3080]	74.5 (11.2)
Pulse pressure, mean (SD), mmHg [*n* = 3080]	65.9 (19.6)
CRP, median (min, max), mg/L [*n* = 2448]	3.3 (0.1–289.0)
CRP > 5 mg/L, *n* (%) [*n* = 2448]	907 (37.1)
Ferritin, mean (SD), μg/L [*n* = 2557]	183.7 (206.5)
Ferritin groups, *n* (%) [*n* = 2557]	
< 100 μg/L	1082 (42.3)
100‒ < 300 μg/L	1042 (40.8)
≥ 300 μg/L	433 (16.9)
Albumin, mean (SD), g/L [*n* = 2865]	42.24 (4.1)
Phosphate, mean (SD), mmol/L [*n* = 2843]	1.15 (0.26)
PTH, median (min, max), ng/L [*n* = 2590]	66 (2–944)
uPCR, median (min, max), g/mol [*n* = 1920]	28 (2–1724)
Lipid-lowering drugs, *n* (%)	1920 (61.3)
Anticoagulants, *n* (%)	1532 (48.9)
ESA dose groups, *n* (%)	
No ESA	2709 (86.5)
ESA, < 40 μg/2 weeks^a^	289 (9.2)
ESA, ≥ 40 μg/2 weeks	134 (4.3)

Baseline was defined as the latest assessment up to 5 days prior to or on the patient entry date. If no such measurement was available, the earliest measurement within 60 days after the patient entry date was utilized

*BMI*, body mass index; *CKD*, chronic kidney disease; *CRP*, C-reactive protein; *CVD*, cardiovascular disease; *DBP*, diastolic blood pressure; *eGFR*, estimated glomerular filtration rate; *ESA*, erythropoiesis-stimulating agent; *Hb*, hemoglobin; *LDL*, low-density lipoprotein; *LVH*, left ventricular hypertrophy; *PTH*, parathyroid hormone; *PVD*, peripheral vascular disease; *SBP*, systolic blood pressure; *SD*, standard deviation; *uPCR*, urinary protein:creatinine ratio

^a^ESA dose cut-off was based on TREAT results, where an average of 20 µg/week seemed a reasonable cut-off for an iron-replete non-dialysis population; units in this table are for darbepoetin alfa

### Time to first major adverse cardiovascular event

**Fig. 1:**
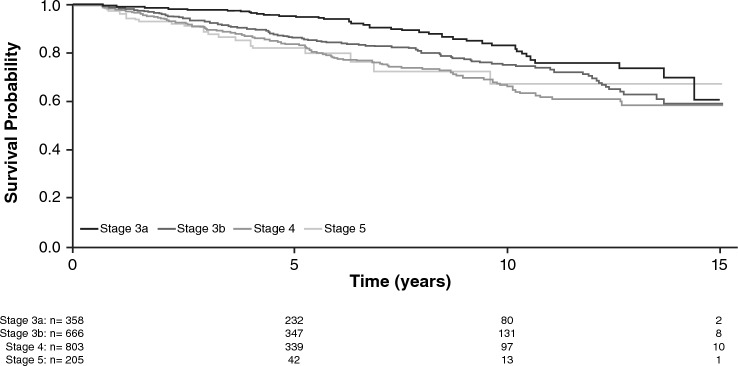
Kaplan–Meier survival estimate of major adverse cardiovascular event (composite of non-fatal myocardial infarction, non-fatal stroke, and/or cardiovascular mortality) in patients with non‒dialysis-dependent chronic kidney disease by chronic kidney disease stage

In the model-building cohort (*n* = 2192), 422 patients (19.3%) experienced a CV-MACE. Mean (SD) time to first CV-MACE was 4.5 (3.4) years (Table S2). The Kaplan–Meier curve for CV-MACE outcomes flattened after 10 years (Fig. 1).

Of the variables that were statistically significant in the univariate analysis (Table S3), a 10-year age increase, history of diabetes or CVD, 5 g/L serum albumin decrease, and ESA treatment were significantly associated with higher CV-MACE risk in the joint final model (Table 2).

**Table 2:** Final joint model results: hazard ratios (95% CIs) for risk factors in patients with non‒dialysis-dependent chronic kidney disease

Dependent variable	Units used to build hazard ratio	Primary endpoint		Secondary endpoints					
		CV-MACE		Any CV event or ACM		CV mortality		ACM	
		Hazard ratio (95% CI)	Wald *P* value	Hazard ratio (95% CI)	Wald *P* value	Hazard ratio (95% CI)	Wald *P* value	Hazard ratio (95% CI)	Wald *P* value
Estimated eGFR at time t	− 5 mL/min/1.73 m^2^	1.03 (0.99–1.07)	0.1199	**1.05 (1.01–1.08)**	**0.003**	1.05 (1.00–1.10)	0.0589	**1.05** **(1.01–1.08)**	**0.011**
Estimated eGFR slope	− 5 mL/min/1.73 m^2^ per year	1.03 (0.73–1.44)	0.8759	1.01 (0.79–1.30)	0.899	0.90 (0.57–1.44)	0.6617	1.03 (0.76–1.39)	0.845
Age, years	+ 10 years	**1.60** **(1.45–1.76)**	** < 0.0001**	**1.89 (1.75–2.04)**	** < 0.001**	**1.73** **(1.55–1.94)**	** < 0.0001**	**2.07** **(1.91–2.25)**	** < 0.001**
Sex	Female vs. male	NA	NA	**0.83 (0.71–0.97)**	**0.018**	NA	NA	NA	NA
Smoking status	Active vs. non-smoker	NA	NA	NA	NA	NA	NA	**1.84 (1.41–2.41)**	** < 0.001**
	Former vs. non-smoker	NA	NA	NA	NA	NA	NA	**1.31 (1.10–1.56)**	**0.002**
Diabetes mellitus	Yes vs. no	**1.39** **(1.13–1.71)**	**0.0016**	**1.23 (1.06–1.43)**	**0.006**	**1.30** **(1.03–1.63)**	**0.0241**	**1.23** **(1.06–1.44)**	**0.008**
CVD	Yes vs. no	**1.71** **(1.39–2.10)**	** < 0.0001**	**1.50 (1.29–1.75)**	** < 0.001**	**1.58** **(1.25–1.98)**	**0.0001**	**1.38** **(1.18–1.62)**	** < 0.001**
Cerebrovascular disease	Yes vs. no	NA	NA	**1.34 (1.08–1.65)**	**0.007**	NA	NA	NA	NA
PVD	Yes vs. no	NA	NA	**1.48 (1.24–1.78)**	** < 0.001**	NA	NA	**1.44** **(1.19–1.73)**	** < 0.001**
Hemoglobin, g/L	+ 10 g/L	1.02 (0.95–1.09)	0.6342	**0.94 (0.89–0.99)**	**0.016**	0.96 (0.89–1.04)	0.3585	**0.90** **(0.85–0.95)**	** < 0.001**
Albumin, g/L	− 5 g/L	**1.20** **(1.05–1.36)**	**0.0061**	**1.19 (1.09–1.30)**	** < 0.001**	**1.17** **(1.01–1.35)**	**0.0341**	**1.16** **(1.05–1.28)**	**0.003**
CRP (log-scale)	× 2	NA	NA			**1.15 (1.08–1.23)**	** < 0.0001**		
CRP > 5 mg/L	> 5 vs. ≤ 5 mg/L	NA	NA	NA	NA	NA	NA	NA	NA
Phosphate (mmol/L)	+ 0.1 mmol/L	NA	NA	**1.05 (1.02–1.08)**	**0.004**	NA	NA	**1.04** **(1.01–1.08)**	**0.021**
PTH (log-scale)	× 2	NA	NA	NA	NA	NA	NA	**1.10** **(1.02–1.19)**	**0.013**
uPCR (log-scale)	× 2	1.06 (0.99–1.13)	0.0901	**1.08 (1.02–1.13)**	**0.005**	**1.08** **(1.00–1.16)**	**0.0465**	**1.08** **(1.03–1.15)**	**0.004**
ESA dose groups	ESA < 40 µg/2 weeks vs. no ESA	**1.61 (1.19–2.17)**	**0.0018**			**2.05 (1.50–2.81)**	** < 0.0001**		
	ESA ≥ 40 µg/2 weeks vs. no ESA	**1.59 (1.01–2.49)**	**0.0435**			1.47 (0.86–2.49)	0.1554		
No. of antihypertensives	+ 1	1.06 (0.99–1.14)	0.1005	NA	NA	**1.15** **(1.06–1.25)**	**0.0010**	NA	NA
Log-ferritin by log-CRP interaction	× 2 and × 2	**1.02 (1.01–1.02)**	**0.0001**			NA	NA		

Bold cells indicate variables that are statistically significant (*P* < 0.05)

*CRP*, C-reactive protein; *CV*, cardiovascular; *CVD*, cardiovascular disease; *eGFR*, estimated glomerular filtration rate; *ESA*, erythropoiesis-stimulating agent; *MACE*, major adverse cardiovascular event (composite of non-fatal myocardial infarction, CV mortality, and/or non-fatal stroke); *NA*, not applicable (i.e., not present in endpoint’s final joint model); *PTH*, parathyroid hormone; *PVD*, peripheral vascular disease; *uPCR*, urinary protein:creatinine ratio

### Time to any cardiovascular event or all-cause mortality

Overall, 792 patients (36.1%) experienced a CV event or died; mean (SD) time to any CV event or ACM was 4.2 (3.14) years. Median time to event was 8.3 years and 6.5 years for patients with stage 4 and stage 5 CKD, respectively. Only 2.0% (16 events) of patients experienced CCF (Table S2).

**Fig. 2:**
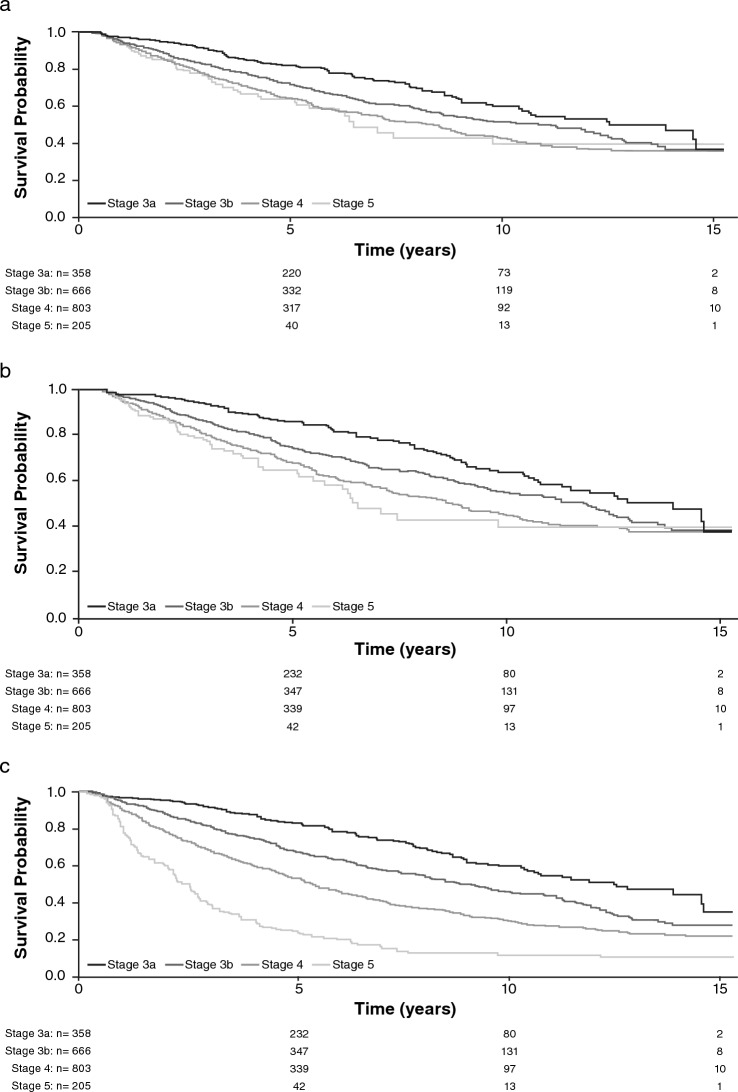
Kaplan–Meier survival estimate of **a** any cardiovascular event and/or all-cause mortality, **b** all-cause mortality, and **c** all-cause mortality or renal replacement therapy in patients with non‒dialysis-dependent chronic kidney disease by chronic kidney disease stage

The risk for any CV event and/or ACM generally increased with increasing CKD stage (Fig. 2a). After 10 years, the prognosis was worse for patients with stage 4 versus stage 5 CKD.

Of the significant variables identified in the univariate analysis (Table S4), a 10-year age increase; history of diabetes, CVD, cerebrovascular disease, or peripheral vascular disease (PVD); 5 mL/min/1.73 m^2^ eGFR decrease; 5 g/L serum albumin decrease; 0.1 mmol/L phosphate increase; and doubling the urinary protein:creatinine ratio significantly increased risk for any CV event or ACM in the final joint model (Table 2). Each hemoglobin level increase of 10 g/L had a statistically significant protective effect, as did female sex.

### Time to individual cardiovascular events

#### Myocardial infarction

Fifty-five patients (2.5%) experienced MI with a mean (SD) time to event of 3.0 (3.28) years (Table S2). Myocardial infarction risk increased with decreasing renal function (Fig. S3).

Statistically significant predictors of increased MI risk in the univariate analysis included 10-year age increase, advanced CKD stage (stage 3b, 4, or 5), history of CVD or diabetes, history of smoking, 5 mL/min/1.73 m^2^ eGFR decrease, halving eGFR from baseline, use of anticoagulants or lipid-lowering drugs, and 10 mmHg increase in pulse pressure (Table S5). Renin-angiotensin blockade was associated with reduced MI risk. No multivariate model for time to first MI could be built as few events occurred.

#### Other individual cardiovascular events

Few patients experienced stroke (2.1%), unstable angina (0.6%), coronary revascularization therapy (0.2%), or CCF (1.0%) (Table S2). Mean (SD) times to event were 3.6 (2.91) years for stroke, 2.9 (3.27) years for unstable angina, 3.8 (4.78) years for coronary revascularization therapy, and 4.2 (3.38) years for CCF. Across individual CV events, ~ 18% of patients were censored for progression to RRT (Table S2).

In univariate analyses, only a few associations between risk factors and individual CV events were observed, including history of CVD for most individual CV events. Due to the limited numbers of events, multivariate models could not be established for these individual CV events.

### Time to cardiovascular mortality

Three hundred and fifty patients (16.0%) died from a CV event with a mean (SD) time to CVM of 5.0 (3.5) years (Table S2). In the final joint model, variables noted to significantly increase CVM risk included 10-year age increase, history of diabetes or CVD, 5 g/L serum albumin decrease, and doubling CRP levels (Table 2).

### Time to all-cause mortality

Seven hundred and forty patients (33.8%) experienced ACM; mean (SD) time to ACM was 4.6 (3.21) years (Table S2). The Kaplan–Meier survival curve flattened after 7.5 years for patients with stage 5 CKD (Fig. 2b), but not when ACM and time to RRT were analyzed together (Fig. 2c).

The final joint model showed that 10-year age increase; history of smoking; history of diabetes, CVD, or PVD; 5 mL/min/1.73 m^2^ eGFR decrease; 5 g/L serum albumin decrease; 0.1 mmol/L phosphate increase; and doubling of parathyroid hormone levels or urinary protein:creatinine ratio significantly increased ACM risk (Table 2). Each 10 g/L increase in hemoglobin levels decreased ACM risk by 10%.

### Time to renal replacement therapy

Overall, 394 patients (18.0%) received RRT; mean (SD) time to RRT was 3.3 (3.04) years (Table S2). The Kaplan–Meier survival curve flattened for patients with stage 4 or 5 CKD (Fig. S4). In the final joint model, halving eGFR, a 10 mmHg increase in systolic blood pressure, and anticoagulant use significantly increased the risk of progression to RRT (Table S6). Female sex and renal diagnoses other than polycystic kidney disease were associated with a significantly decreased risk of progression to RRT.

### Post hoc analysis: time to first major adverse cardiovascular event

In the post hoc analysis in which MACE was defined to include ACM rather than CVM, 780 patients (35.6%) experienced MACE. Mean (SD) time to event was 4.3 (3.1) years (Table S2). The Kaplan–Meier curve for MACE outcomes flattened after 10 years (Fig. S5).

Of the variables that were statistically significant in the univariate analysis (Table S7), 10-year age increase; history of diabetes, CVD, cerebrovascular disease, or PVD; 5 mL/min/1.73 m^2^ eGFR decrease; 5 g/L serum albumin decrease; and 0.1 mmol/L phosphate increase were significantly associated with higher risk of MACE in the joint final model (Table S8). Female sex and 10 g/L increase in hemoglobin levels each had a protective effect.

### Subgroup analyses

For the stratified subgroup analyses, 37% of the sample had CRP levels > 5 mg/L; 42% of the sample had ferritin levels < 100 µg/L, 41% between 100 and 300 µg/L, and 17% ≥ 300 µg/L.

More than 50% of patients in the CRP > 5 mg/L subgroup and more than 40% in the ferritin ≥ 300 µg/L subgroup experienced MACE (Table S9). For all other subgroups, the percentage of patients experiencing MACE was between 31 and 38%. The hazard ratios were generally comparable across these different subgroups. Log-ferritin by log-CRP interaction had a significant impact on the MACE risk in two subgroups (CRP > 5 mg/L and ferritin ≥ 300 µg/L).

Approximately 19% of the model-building cohort (*n* = 422) had a CV-MACE, with a mean follow-up time of 5.6 years (Table S10). Event rates were slightly higher (21%) in patients with non-missing CRP (*n*/*N* = 351/1710) or ferritin (366/1767) values. CV-MACE events were observed in 28% and 23% of patients in the CRP > 5 mg/L and ferritin ≥ 300 µg/L subgroups, respectively. Although not all variables reached significance, the hazard ratios were comparable between the CRP and ferritin subgroups for each eGFR decrease of 5 mL/min/1.73 m^2^, aging, history of diabetes or CVD, decrease in albumin levels, urinary protein:creatinine ratio, and each hemoglobin increase of 10 g/L. Although any ESA treatment was associated with a significantly increased risk of CV-MACE in the total cohort, only ESA dose < 40 µg per 2 weeks was associated with a significantly increased risk of CV-MACE in the CRP > 5 mg/L and ferritin ≥ 300 µg/L subgroups.

### Sensitivity analyses

In the sensitivity analysis for time to first CV-MACE without forcing the retention of clinically important variables, hemoglobin and urinary protein:creatinine ratio were dropped from the final model. The resulting risk estimates were similar to the primary model (Table S11).

In the second sensitivity analysis, urinary protein:creatinine ratio was dropped from the model. The magnitude of the risks and majority of risk factors in this analysis were similar to the primary model (Table S12).

### Model validation

The AUCs show the model adequately predicts the cumulative risk for time to first CV-MACE (Fig. S6). AUCs across the remaining study endpoints were generally ≥ 0.80.

## Discussion

Patients with NDD-CKD were at an increased risk for CV events. Significantly higher risks of CV-MACE, CVM, and all other individual CV and mortality outcomes were associated with increasing age, a 5 g/L serum albumin decrease, and history of diabetes or CVD. Erythropoiesis-stimulating agent treatment was also associated with significantly higher risks of CV-MACE and CVM, which requires further investigation. Each 5 mL/min/1.73 m^2^ decrease in eGFR and doubling of the urinary protein:creatinine ratio was associated with an increased risk of all outcomes except CV-MACE. Doubling CRP levels and antihypertensive use were also associated with increased CVM risk; a history of smoking and doubling parathyroid hormone levels were also associated with ACM. These findings are consistent with results from other studies in NDD-CKD [15–17].

Anticoagulation use was associated with progression to RRT. Although precise causes for this cannot be ascertained, potential explanations are that atrial fibrillation prevalence increases with worsening CKD stage, and vitamin K antagonists (e.g., warfarin) may precipitate anticoagulant nephropathy.

After 10 years, patients with stage 4 versus 5 CKD had a worse prognosis for any CV event and/or ACM. This may have been due to low patient numbers at later time points, but is most likely due to competing risks: fewer patients with stage 4 (vs. stage 5) CKD would be expected to progress to RRT in this time frame; therefore, this analysis is more likely to capture patients with stage 4 CKD who die. The Kaplan–Meier curve for time to RRT flattened over time for patients with stage 4 or 5 CKD, perhaps due to a mix of no event and mortality censoring or survivor bias. The Kaplan–Meier curve for CV-MACE outcomes also flattened after 10 years, perhaps due to survivor bias (many SKS patients were recruited within the past 10 years, therefore few would have had > 10 years of follow-up).

In our analysis, a 10 g/L increase in hemoglobin levels relative to another patient was associated with decreased risk of any CV event and ACM combined and decreased risk of ACM alone in a population in which most patients (86.5%) were not receiving ESAs. Although we did not capture iron replacement therapy in this analysis, the mean hemoglobin level was 123.6 g/L and mean ferritin level was 183.7 μg/L. Anemia of CKD is associated with increased risk of CVD, impaired activity and work productivity, and significant decreases in patients’ quality of life (QOL) [9, 18].

Previous studies have reported on CV events or risks of CV events associated with anemia and/or ESAs in patients with NDD-CKD. The FIND-CKD trial found no difference in CV events between patients receiving intravenous ferric carboxymaltose and oral iron [19]. In the TREAT trial, use of darbepoetin alfa did not reduce risks of death, CV events, or end-stage renal disease compared with placebo; did not improve QOL; was associated with an increased risk of stroke [20]. In a real-world European study, patients with NDD-CKD and anemia (vs those without anemia) had a significantly higher number of concomitant CV conditions (1.27 vs. 0.95; *P* < 0.001) and having CV conditions was associated with significantly reduced QOL (*P* = 0.028) and work productivity (*P* = 0.032) [21].

The results of our hypothesis-generating research can be used to guide the development of future CKD-specific CV risk equations. The risk factors identified in our analysis of SKS patient data could be used as inputs in future prognostic models. For example, Grams et al. generated a risk calculator for RRT, nonfatal CVD events, and death (http://ckdpcrisk.org/lowgfrevents/) using a Markov model based on data from cohorts of patients participating in the international Chronic Kidney Disease Prognosis Consortium [22]. Similarly, Schlackow et al. developed a Markov model using individual patient data from the SHARP study [23].

The main limitations of this analysis were related to incompleteness of data (e.g., unknown CKD diagnosis date, not considering albuminuria), which represents real-world clinical practice. The frequency of CCF events was unexpectedly low (2.0%), considering the number of patients with a history of CVD at baseline (34.6%), perhaps due to difficulties in recording this endpoint correctly or flaws in its designation at follow-up.

We used a robust multiple imputation method to generate values for covariate data with missing values, but the underlying ‘missing at random’ assumption is untestable. Residual confounding may have occurred despite using multivariate statistical techniques to adjust for confounding covariates, as additional confounding factors may not have been collected. Likewise, errors may have occurred in the classification of patients with respect to confounding variables. However, these limitations were mitigated by the large sample size, long follow-up duration, and robust joint modeling approach, which can be replicated in future studies.

Our results may not be generalizable to broader NDD-CKD populations in the UK or elsewhere. Approximately 18% of Salford center patients receiving dialysis are non-white [24], whereas in our SKS analysis of patients with NDD-CKD, this figure was only 4%. This indicates the difficulty/barriers in recruiting patients from other ethnic backgrounds into research.

Our results show that patients with NDD-CKD were at an increased risk of any CV event, ACM, and RRT. In addition to traditional CV risk factors (age, diabetes), risk factors such as decreases in eGFR values and serum albumin and history of CVD, PVD, or cerebrovascular disease were associated with increased mortality and CV event risk in this population. Novel biomarkers, while not explicitly evaluated in this study, may be appropriate to incorporate into future models [25]. Event rates were slightly higher in the ferritin subgroup analysis compared with the total study sample analysis. The non-traditional risk factors identified in our hypothesis-generating analysis may be more specific to patients with NDD-CKD and could be used as inputs in the future development of a new CKD-specific CV risk equation that is more tailored to the CKD population than the Framingham equation or other general risk scores. Such an equation is needed to address the high burden of CVD in patients with NDD-CKD, considering the impact of each factor on CV risk.

## Supplementary Information

Below is the link to the electronic supplementary material.

Supplementary file1 (DOCX 543 kb)

## Data Availability

Researchers may request access to anonymized participant level data, trial level data and protocols from Astellas sponsored clinical trials at www.clinicalstudydatarequest.com. For the Astellas criteria on data sharing see: https://clinicalstudydatarequest.com/Study-Sponsors/Study-Sponsors-Astellas.aspx.
