# Supplementary material for: Clinical factors for predicting cardiovascular risk, need for renal replacement therapy, and mortality in patients with non–dialysis-dependent stage 3–5 chronic kidney disease from the Salford Kidney Study
Source: J Nephrol. 2023 Jun 8;36(6):1639–49. doi: 10.1007/s40620-023-01626-8 (PMC10393868; doi:10.1007/s40620-023-01626-8)
Supplement: Supplementary file 1 — Supplementary file1 (DOCX 543 kb) [file 40620_2023_1626_MOESM1_ESM.docx]

**Clinical factors for predicting cardiovascular risk, renal replacement therapy, and mortality in patients with non–dialysis-dependent stage 3-5 chronic kidney disease from the Salford Kidney Study**

Ana Filipa Alexandre, Matthias Stoelzel, Amit Kiran, Alberto Garcia Hernandez, Antonia Morga, Philip A. Kalra

# SUPPLEMENTARY MATERIAL

***Journal of Nephrology***

**Correspondence to:** Ana Filipa Alexandre, Pricing and Market Access, Santen Pharmaceutical, Alpha Tower, De Entree 11-97, 1101 BH Amsterdam, Netherlands; E-mail: anafalexandre@gmail.com; Telephone: +31628850790

## Online Resource 1: Additional Information on Study Methods

## Supplemental Tables

## Supplemental Figures

## Online Resource 1: Additional Information on Study Methods

***Treatment information***

Between 2002 and 2016, the center’s approach to management of anemia did not change. Those patients treated with an erythropoiesis-stimulating agent (ESA) had target hemoglobin of <120 g/L. If patients with anemia were iron deficient, they received intravenous iron to maintain serum ferritin levels >100 µg/L. Management of chronic kidney disease (CKD) mineral bone disease in this period involved use of standard phosphate binders where appropriate and alfacalcidol. There was no routine measurement of vitamin D levels or treatment with cholecalciferol. Patients at increased risk of cardiac events (e.g., patients with diabetes or established cardiovascular disease) were treated with statins.

### Data collection

Patient demographic data, comorbidities, and concomitant medication use were recorded at study entry (baseline). Routine blood and urine samples were obtained for laboratory testing and recorded at baseline and at annual visits. Demographic data, cardiovascular (CV) events, and details of any other hospitalizations were also captured at annual visits.

Cardiovascular events were adjudicated centrally by the research team; other events were recorded from patient-provided information or abstracted from discharge documents. Patient-reported history of CV events were reviewed and verified by three research team physicians to decrease recall bias.

To ensure relevant CV events were not missed, a list of acceptable synonyms (e.g., stroke and cerebrovascular accident) was used. Initially, Office of National Statistics (ONS) cause-of-death data were available only through December 2013. Therefore, the cause of death for deaths occurring from January 2014 onward was obtained, where possible, from the electronic patient record. However, the ONS later provided cause-of-death data for the full observation period.

### Statistical analysis

Baseline variables related to comorbidities were set to ‘No’ if their raw value was ‘No’, ‘Missing’, ‘Unobtainable’, or blank in the database. Composite variables at baseline were set to ‘No’ if none of the components had a value of ‘Yes’. A missing admission date (event date for CV events) was imputed by the midpoint of the two follow-up visits between which the event was first recorded. In analyses in which weight, estimated glomerular filtration rate (eGFR), CKD stage, albumin, hemoglobin, hemoglobin A1c, C‑reactive protein (CRP), parathyroid hormone, urinary protein:creatinine ratio, or ferritin covariate values were missing, a multiple imputation procedure (10 datasets) was used to replace missing data. The imputation model included weight, sex, height, and the post-baseline observations of the variable to impute. Other missing values were not imputed.

In univariate time-to-event analyses, continuous parameters were modeled either untransformed or log-transformed, assuming a linear relationship between continuous covariates and the log-hazard function. Univariate analyses were performed to determine whether a baseline clinical or laboratory parameter predicted a study outcome. Variables identified as risks using a significance level of P≤0.1 and clinically important variables (i.e., age, race/ethnicity, sex, smoking history, history of cardiovascular disease (CVD), diabetes, systolic blood pressure, body mass index, CKD stage, hemoglobin, hemoglobin A1c, high-density lipoprotein cholesterol, low-density lipoprotein cholesterol, eGFR, urine protein, albumin, sodium, potassium, erythropoiesis-stimulating agent [ESA] use) were included in a fully parametric multivariable proportional hazards (PH) model followed by a backward selection procedure, guided by the Bayesian information criterion (BIC), to identify a provisional survival model. A limited number of variables considered essential for a CV risk equation (e.g., age, history of CVD or diabetes, albumin, eGFR, hemoglobin, urine protein) were retained in the final multivariable PH model even if the BIC suggested they could be dropped. The selected clinical and laboratory parameters and concomitant medication variables used were chosen based on well-known risk factors established for CV risk equations [1]. The resulting parametric survival model was fitted with a random intercepts and slope model for eGFR in a joint model [2]. Multivariable modeling for secondary endpoints was only performed if at least three events occurred per parameter in the model (e.g., a model with 10 risk factors required 30 events).

The associations of risk factors with outcomes were investigated in a joint model that combined a repeated measures model for eGFR and the provisional multivariable PH model for risk factors at baseline.

Two sensitivity analyses were conducted: repetition of the backward selection procedure (i) without clinically important variables and (ii) using Akaike’s information criterion for clinically important variables and BIC for other variables.

### Post hoc analyses

When the initial analysis was conducted, cause-of-death data were not available for 54.3% of patients who died during the observation period. When full cause-of-death data became available in February 2021, the major adverse CV event (CV-MACE) and CV mortality (CVM) analyses were re-run using the complete dataset. A new analysis was also conducted in which MACE was redefined to include ACM (i.e., MACE = non-fatal myocardial infarction, non-fatal stroke, death due to any cause) rather than CV death.

When the post hoc CV-MACE and CVM analyses were performed, new subgroup analyses were added to explore risk factors for CV-MACE and MACE by baseline CRP (>5 mg/L, ≤5 mg/L) and ferritin (<100 µg/L, ≥100 to <300 µg/L, ≥300 µg/L) levels. The post hoc analyses were conducted as described above for the main analysis, with updated risk equations for the CV-MACE and MACE outcomes.

**REFERENCES**

1. Major RW, Cheng MRI, Grant RA*,* et al (2018) Cardiovascular disease risk factors in chronic kidney disease: a systematic review and meta-analysis. PLoS One 13:e0192895. https://doi.org/10.1371/journal.pone.0192895

2. Diggle PJ, Sousa I, Chetwynd AG (2008) Joint modelling of repeated measurements and time-to-event outcomes: the fourth Armitage lecture. Stat Med 27:2981-2998. https://doi.org/10.1002/sim.3131

## Supplemental Tables

Table S1. Clinical and laboratory parameters used as independent variables

| **Clinical parameters** | **Laboratory parameters** | **Concomitant medication use** |
| --- | --- | --- |
| - Age - Race/ethnicity - Sex - Smoking history - Presence of diabetes - History of CV or cerebrovascular diseases - Systolic blood pressure - Diastolic blood pressure - Pulse pressure - Peripheral vascular disease - Left ventricular hypertrophy - Body mass index - History of anemia - CKD stage - Primary renal disease - Malignancy - Presence of inflammation | - Hemoglobin (continuous and categorical) - Hematocrit - Total cholesterol (continuous and categorical) - Low-density lipoprotein cholesterol - High-density lipoprotein cholesterol - Triglycerides - Serum ferritin (continuous and categorical) - Transferrin saturation - Hemoglobin A1c - Creatinine - eGFR (including change over time) - Sodium - Potassium - Albumin - Phosphate - Corrected calcium - Intact parathyroid hormone - C-reactive protein - Urinary protein: creatinine ratio | - Erythropoiesis-stimulating agents - Renin-angiotensin blockades - Anti-hypertensive treatment (angiotensin-converting-enzyme inhibitors and angiotensin II receptor blockers vs. other antihypertensive medication) - Lipid-lowering treatment - Anticoagulants |

Abbreviations: CKD, chronic kidney disease; CV, cardiovascular; eGFR, estimated glomerular filtration rate.

Table S2. Description of events and times to events in patients with non‒dialysis-dependent chronic kidney disease^a^

| **Variable** | **Primary endpoint** |  | **Secondary endpoints** | | | | | | | | | **Exploratory endpoint** |
| --- | --- | --- | --- | --- | --- | --- | --- | --- | --- | --- | --- | --- |
|  | **First CV-MACE^b^** | **First MACE^c^** | **Any CV event or all-cause mortality** | **Individual CV event** | | | | | **CV mortality** | | **All-cause mortality** | **First  RRT** |
|  |  |  |  | **First MI** | **First stroke** | **First UA** | **First CRT** | **First CCF** |  |  |  |  |
| Event, n (%) | 422 (19.3) | 780 (35.6) | 792 (36.1) | 55 (2.5) | 45 (2.1) | 13 (0.6) | 4 (0.2) | 21 (1.0) | | 350 (16.0) | 740 (33.8) | 394 (18.0) |
| Cause of event, n (% of events) |  |  |  |  |  |  |  |  | |  |  |  |
| MI | 53 (12.6) | 53 (6.8) | 50 (6.3) | 54 (98.2) | NA | NA | NA | NA | | NA | NA | NA |
| Stroke | 44 (10.4) | 44 (5.6) | 44 (5.6) | NA | 45 (100) | NA | NA | NA | | NA | NA | NA |
| UA | NA | NA | 11 (1.4) | NA | NA | 13 (100) | NA | NA | | NA | NA | NA |
| CRT | NA | NA | 3 (0.4) | NA | NA | NA | 3 (75.0) | NA | | NA | NA | NA |
| CCF | NA | NA | 16 (2.0) | NA | NA | NA | NA | 21 (100) | | NA | NA | NA |
| MI and CRT | 1 (0.2) | 1 (0.1) | 1 (0.1) | 1 (1.8) | NA | NA | 1 (25.0) | NA | | NA | NA | NA |
| Unknown cause of death | NA | NA | 370 (46.7) | NA | NA | NA | NA | NA | | NA | 402 (54.3) | NA |
| CV death | 324 (76.8) | 324 (41.5) | 88 (11.1) | NA | NA | NA | NA | NA | | 350 (100) | 103 (13.9) | NA |
| Non-CV death | NA | 358 (45.9) | 209 (26.4) | NA | NA | NA | NA | NA | | NA | 235 (31.8) | NA |
| RRT | NA | NA | NA | NA | NA | NA | NA | NA | | NA | NA | 394 (100.0) |
| Censored, n (%) | 1770 (80.8) | 1412 (64.5) | 1400 (63.9) | 2137 (97.5) | 2147 (98.0) | 2179 (99.4) | 2188 (99.8) | 2171 (99.0) | | 1842 (84.0) | 1452 (66.2) | 1798 (82.0) |
| Cause of censoring, n (% of censorings) |  |  |  |  |  |  |  |  |  | |  |  |
| Unknown cause of death | 89 (5.0) | NA | NA | 406 (19.0) | 409 (19.1) | 419 (19.2) | 421 (19.2) | 416 (19.2) | 96 (5.2) | | NA | 421 (23.4) |
| CV death | NA | NA | NA | 99 (4.6) | 96 (4.5) | 102 (4.7) | 103 (4.71) | 98 (4.51) | NA | | NA | 103 (5.7) |
| Non-CV death | 274 (15.5) | NA | NA | 223 (10.4) | 224 (10.4) | 233 (10.7) | 235 (10.7) | 229 (10.6) | 299 (16.2) | | NA | 235 (13.1) |
| RRT | 386 (21.8) | 386 (27.3) | 381 (27.2) | 390 (18.3) | 391 (18.2) | 390 (17.9) | 394 (18.0) | 395 (18.2) | 395 (21.4) | | 395 (27.2) | NA |
| Database extract | 1021 (57.7) | 1026 (72.7) | 1019 (72.8) | 1019 (47.7) | 1027 (47.8) | 1035 (47.5) | 1035 (47.3) | 1033 (47.6) | 1052 (57.1) | | 1057 (72.8) | 1039 (57.8) |
| Time to event, mean (SD), years | 4.5 (3.4) | 4.3 (3.15) | 4.2 (3.14) | 3.0 (3.28) | 3.6 (2.91) | 2.9 (3.27) | 3.8 (4.78) | 4.2 (3.38) | 5.0 (3.5) | | 4.6 (3.21) | 3.3 (3.04) |
| Time to censoring, mean (SD), years | 5.8 (4.02) | 6.3 (4.15) | 6.2 (4.14) | 5.7 (3.9) | 5.7 (3.94) | 5.7 (3.94) | 5.7 (3.94) | 5.7 (3.95) | 5.9 (4.0) | | 6.3 (4.15) | 6.3 (3.92) |

^a^ Analysis performed on the training dataset (70% of the total analysis set; 2192/3132).

^b^ Composite of non-fatal MI, non-fatal stroke, or CV death.

^C^ Composite of non-fatal MI, non-fatal stroke, or all-cause mortality.

CCF, congestive cardiac failure; CRT, coronary revascularization therapy; CV, cardiovascular; MACE, major adverse cardiovascular event (composite of non-fatal MI, CV mortality, and/or non-fatal stroke); MI, myocardial infarction; n, number of valid observations; NA, not applicable; RRT, renal replacement therapy; SD, standard deviation; UA, unstable angina.

Table S3. Time to first CV-MACE: univariate analysis results (proportional hazards approach)

| **Dependent variable** | **n** | **LRT** | **LRT  P value** | **ΔBIC^a^** | **Units used to build hazard ratio** | **Hazard ratio** | **95% CI** | **Wald  P value** |
| --- | --- | --- | --- | --- | --- | --- | --- | --- |
| **Renal diagnosis** | **2192** | **144.3** | **<0.0001** | **-95.9** | **Diabetic nephropathy vs. PKD** | 4.81 | 2.24-10.33 | **<0.0001** |
|  |  |  |  |  | **Hypertensive nephrosclerosis vs. PKD** | 3.28 | 1.50-7.18 | **0.0029** |
|  |  |  |  |  | **Renovascular disease vs. PKD** | 6.31 | 2.88-13.82 | **<0.0001** |
|  |  |  |  |  | Glomerulonephritis vs. PKD | 1.57 | 0.70-3.55 | 0.2730 |
|  |  |  |  |  | Chronic pyelonephritis vs. PKD | 0.99 | 0.39-2.51 | 0.9809 |
|  |  |  |  |  | **Unknown vs. PKD** | 3.49 | 1.60-7.59 | **0.0016** |
|  |  |  |  |  | Others vs. PKD | 2.08 | 0.95-4.55 | 0.0679 |
|  |  |  |  |  | Missing vs. PKD | 0.39 | 0.11-1.34 | 0.1353 |
| **eGFR (mL/min/1.73 m^2^)** | **1498** | **54.0** | **<0.0001** | **-48.3** | **-5 mL/min/1.73 m^2^** | **1.17** | **1.12-1.22** | **<0.0001** |
| **eGFR (log-scale)** | **1498** | **52.1** | **<0.0001** | **-46.4** | **÷2** | **1.91** | **1.61-2.28** | **<0.0001** |
| **CKD stage** | **1498** | **56.2** | **<0.0001** | **-39.2** | **Stage 3b vs. stages 1, 2, and 3a** | **2.17** | **1.52-3.10** | **<0.0001** |
|  |  |  |  |  | **Stage 4 vs. stages 1, 2, and 3a** | **3.13** | **2.22-4.42** | **<0.0001** |
|  |  |  |  |  | **Stage 5 vs. stages 1, 2, and 3a** | **4.05** | **2.42-6.79** | **<0.0001** |
| **Age (years)** | **2192** | **197.5** | **<0.0001** | **-191.4** | **+10 years** | **1.76** | **1.61-1.92** | **<0.0001** |
| Sex | 2192 | 3.1 | 0.0783 | 3.0 | Female vs. male | 0.84 | 0.69-1.02 | 0.0821 |
| Ethnic group | 2189 | 6.4 | 0.0937 | 11.7 | Asian vs. white | 0.50 | 0.22-1.12 | 0.0933 |
|  |  |  |  |  | Black vs. white | 0.81 | 0.26-2.52 | 0.7152 |
|  |  |  |  |  | Other vs. white | 0.00 | 0.00 to >99 | 0.9813 |
| **Smoking status** | **2192** | **19.0** | **<0.0001** | **-6.9** | Active smoker vs. non-smoker | 1.12 | 0.79-1.58 | 0.5332 |
|  |  |  |  |  | **Former smoker vs. non-smoker** | **1.58** | **1.27-1.96** | **<0.0001** |
| **Diabetes mellitus** | **2192** | **39.8** | **<0.0001** | **-33.7** | **Yes vs. no** | **1.89** | **1.56-2.30** | **<0.0001** |
| **CVD** | **2192** | **113.6** | **<0.0001** | **-107.5** | **Yes vs. no** | **2.86** | **2.36-3.47** | **<0.0001** |
| **Cerebrovascular disease** | **2192** | **24.2** | **<0.0001** | **-18.2** | **Yes vs. no** | **2.12** | **1.61-2.79** | **<0.0001** |
| **PVD** | **2192** | **22.5** | **<0.0001** | **-16.4** | **Yes vs. no** | **1.82** | **1.44-2.30** | **<0.0001** |
| **LVH** | **2192** | **4.0** | **0.0455** | **2.1** | **Yes vs. no** | **2.22** | **1.10-4.47** | **0.0255** |
| **SBP (mmHg)** | **2161** | **13.8** | **0.0002** | **-7.8** | **+10 mmHg** | **1.09** | **1.04-1.14** | **0.0002** |
| **DBP (mmHg)** | **2152** | **14.6** | **0.0001** | **-8.6** | **+10 mmHg** | **0.84** | **0.77-0.92** | **0.0002** |
| **Pulse pressure (mmHg)** | **2152** | **41.5** | **<0.0001** | **-35.5** | **+10 mmHg** | **1.18** | **1.12-1.23** | **<0.0001** |
| Weight (kg) | 1825 | 0.1 | 0.7518 | 5.8 | +10 kg | 0.99 | 0.94-1.05 | 0.8285 |
| Height (m) | 2135 | 0.4 | 0.5271 | 5.6 | +10 cm | 0.97 | 0.88-1.07 | 0.5195 |
| BMI (kg/m^2^) | 1790 | 0.0 | 1.000 | 5.8 | +5 kg/m^2^ | 0.99 | 0.91-1.08 | 0.8728 |
| **Hemoglobin (g/L)** | **1998** | **21.1** | **<0.0001** | **-15.1** | **+10 g/L** | **0.87** | **0.82-0.92** | **<0.0001** |
| **Hemoglobin <100 g/L** | **1998** | **9.5** | **0.0021** | **-3.5** | **<100 vs. ≥100 g/L** | **1.90** | **1.31-2.76** | **0.0008** |
| **Hemoglobin categories** | **1998** | **24.4** | **<0.0001** | **-0.3** | **<100 vs. ≥130 g/L** | **2.38** | **1.60-3.54** | **<0.0001** |
|  |  |  |  |  | **100-110 vs. ≥130 g/L** | **1.64** | **1.20-2.25** | **0.0019** |
|  |  |  |  |  | **110-120 vs. ≥130 g/L** | **1.53** | **1.18-1.99** | **0.0016** |
|  |  |  |  |  | **120-130 vs. ≥130 g/L** | **1.30** | **1.00-1.69** | **0.0500** |
| **CRP (mg/L)** | **1710** | **22.6** | **<0.0001** | **-16.7** | **+5 mg/L** | **1.07** | **1.05-1.09** | **<0.0001** |
| **CRP (log-scale)** | **1710** | **50.2** | **<0.0001** | **-44.3** | **x2** | **1.25** | **1.17-1.33** | **<0.0001** |
| **CRP >5 mg/L** | **1710** | **48.0** | **<0.0001** | **-42.1** | **>5 vs. ≤5 mg/L** | **2.12** | **1.72-2.61** | **<0.0001** |
| **LDL cholesterol (calculated; mmol/L)** | **1517** | **17.6** | **<0.0001** | **-11.9** | **+1 mol/L** | **0.80** | **0.71-0.89** | **<0.0001** |
| LDL cholesterol groups | 2192 | 16.3 | 0.0003 | -4.2 | <3 vs. ≥3 mol/L | 1.44 | 1.09-1.90 | **0.0101** |
|  |  |  |  |  | Missing vs. ≥3 mol/L | 0.95 | 0.70-1.29 | 0.7447 |
| Triglycerides (mmol/L) | 92 | 0.5 | 0.4795 | 2.9 | +1 mol/L | 0.91 | 0.70-1.20 | 0.5136 |
| Triglycerides groups | 2192 | 7.8 | 0.0202 | 4.4 | ≤2.3 vs. >2.3 mol/L | 1.58 | 0.64-3.86 | 0.3192 |
|  |  |  |  |  | Missing vs. >2.3 mol/L | 0.84 | 0.37-1.87 | 0.6630 |
| **Total cholesterol (mmol/L)** | **1861** | **21.0** | **<0.0001** | **-15.0** | **+1 mol/L** | **0.81** | **0.74-0.89** | **<0.0001** |
| **Total cholesterol >5 mmol/L** | **1861** | **11.4** | **0.0007** | **-5.5** | **≥5 vs. <5 mol/L** | **0.69** | **0.55-0.86** | **0.0010** |
| **Hematocrit (ratio)** | **1998** | **14.7** | **0.0001** | **-8.7** | **+0.1 L/L** | **0.66** | **0.54-0.82** | **0.0001** |
| HDL cholesterol (mmol/L) | 1521 | 1.6 | 0.2059 | 4.1 | +0.5 mol/L | 0.93 | 0.82-1.04 | 0.2066 |
| **Iron saturation (%)** | **1771** | **7.1** | **0.0077** | **-1.3** | **+10%** | **0.87** | **0.79-0.97** | **0.0091** |
| **Albumin (g/L)** | **2005** | **20.3** | **<0.0001** | **-14.3** | **-5 g/L** | **1.26** | **1.15-1.39** | **<0.0001** |
| **Phosphate (mmol/L)** | **1992** | **7.2** | **0.0073** | **-1.2** | **+0.1 mmol/L** | **1.05** | **1.01-1.09** | **0.0066** |
| Corrected calcium (mmol/L) | 2001 | 2.8 | 0.0943 | 3.2 | +0.1 mmol/L | 0.94 | 0.87-1.01 | 0.0985 |
| **PTH (ng/L)** | **1808** | **22.8** | **<0.0001** | **-16.9** | **+100 ng/L** | **1.33** | **1.20-1.48** | **<0.0001** |
| **PTH (log-scale)** | **1808** | **28.8** | **<0.0001** | **-22.9** | **x2** | **1.28** | **1.17-1.40** | **<0.0001** |
| uPCR (g/mol) | 1349 | 1.3 | 0.2542 | 4.3 | +100 g/mol | 1.04 | 0.97-1.12 | 0.2342 |
| **uPCR (log-scale)** | **1349** | **10.2** | **0.0014** | **-4.6** | **x2** | **1.11** | **1.04-1.18** | **0.0012** |
| **Sodium (mmol/L)** | **2007** | **6.1** | **0.0135** | **-0.0** | **+5 mmol/L** | **0.82** | **0.71-0.96** | **0.0126** |
| Potassium (mmol/L) | 1990 | 0.6 | 0.4386 | 5.4 | +5 mmol/L | 1.39 | 0.62-3.12 | 0.4255 |
| **Ferritin (µg/L)** | **1767** | **8.1** | **0.0044** | **-2.2** | **+100 µg/L** | **1.06** | **1.03-1.10** | **0.0009** |
| **Ferritin (log-scale)** | **1767** | **8.2** | **0.0042** | **-2.3** | **x2** | **1.11** | **1.03-1.19** | **0.0045** |
| **Ferritin groups** | 1767 | 12.8 | 0.0017 | -0.9 | 100 to <300 vs. <100 µg/L | 1.12 | 0.89-1.41 | 0.3304 |
|  |  |  |  |  | **≥300 vs. <100 µg/L** | **1.72** | **1.29-2.28** | **0.0002** |
| HbA1c (mmol/mol) | 343 | 2.6 | 0.1069 | 1.8 | +10 mmol/mol | 1.09 | 0.99-1.21 | 0.0911 |
| **HbA1c groups** | 2192 | 19.6 | 0.0006 | 4.6 | 41 to <48 vs. ≤41 mmol/mol | 1.04 | 0.46-2.35 | 0.9291 |
|  |  |  |  |  | **48 to <75 vs. ≤41 mmol/mol** | **2.09** | **1.10-3.95** | **0.0236** |
|  |  |  |  |  | >75 vs. ≤41 mmol/mol | 2.07 | 0.97-4.43 | 0.0604 |
|  |  |  |  |  | Missing vs. ≤41 mmol/mol | 1.07 | 0.60-1.91 | 0.8107 |
| **ESA dose groups** | **2192** | **26.8** | **<0.0001** | **-14.6** | **ESA, <40 µg/2 weeks vs. no ESA** | **2.14** | **1.61-2.84** | **<0.0001** |
|  |  |  |  |  | **ESA, ≥40 µg/2 weeks vs. no ESA** | **1.71** | **1.12-2.62** | **0.0124** |
| Renin-angiotensin blockade | 2192 | 1.3 | 0.2542 | 4.8 | Yes vs. no | 0.89 | 0.73-1.09 | 0.2587 |
| **Number of antihypertensives** | **2166** | **32.0** | **<0.0001** | **-26.0** | **+1** | **1.18** | **1.10-1.25** | **<0.0001** |
| **Lipid-lowering drugs** | **2192** | **25.1** | **<0.0001** | **-19.0** | **Yes vs. no** | **1.68** | **1.36-2.07** | **<0.0001** |
| **Anticoagulants** | **2192** | **64.0** | **<0.0001** | **-57.9** | **Yes vs. no** | **2.22** | **1.82-2.72** | **<0.0001** |

**Bold** and shaded rows indicate variables that are statistically significant (P<0.05).

^a^ Negative ΔBIC means that the variable improves the BIC.

BIC, Bayesian information criterion; BMI, body mass index; CKD, chronic kidney disease; CI, confidence interval; CRP, C-reactive protein; CVD, cardiovascular disease; DBP, diastolic blood pressure; eGFR, estimated glomerular filtration rate; ESA, erythropoiesis-stimulating agent; Hb, hemoglobin; HDL, high-density lipoprotein; LDL, low-density lipoprotein; LRT, likelihood ratio test; LVH, left ventricular hypertrophy; n, number of valid observations; PKD, polycystic kidney disease; PTH, parathyroid hormone; PVD, peripheral vascular disease; SBP, systolic blood pressure; SD, standard deviation; uPCR, urinary protein:creatinine ratio.

Table S4. Time to first cardiovascular event or all-cause mortality: univariate analysis results (proportional hazards approach)

| **Dependent variable** | **n** | **LRT** | **LRT  P value** | **ΔBIC^a^** | **Units used to build hazard ratio** | **Hazard ratio** | **95% CI** | **Wald  P value** |
| --- | --- | --- | --- | --- | --- | --- | --- | --- |
| **Renal diagnosis** | 2192 | 233.9 | <0.0001 | -180.6 | **Diabetic nephropathy vs. PKD** | **5.69** | **3.02-10.72** | **<0.0001** |
|  |  |  |  |  | **Hypertensive nephrosclerosis vs. PKD** | **4.26** | **2.24-8.12** | **<0.0001** |
|  |  |  |  |  | **Renovascular disease vs. PKD** | **8.50** | **4.46-16.19** | **<0.0001** |
|  |  |  |  |  | **Glomerulonephritis vs. PKD** | **2.22** | **1.15-4.29** | **0.0182** |
|  |  |  |  |  | Chronic pyelonephritis vs. PKD | 1.79 | 0.88-3.65 | 0.1083 |
|  |  |  |  |  | **Unknown vs. PKD** | **4.40** | **2.31-8.35** | **<0.0001** |
|  |  |  |  |  | **Others vs. PKD** | **2.86** | **1.50-5.45** | **0.0014** |
|  |  |  |  |  | Missing vs. PKD | 0.57 | 0.23-1.44 | 0.2360 |
| **eGFR (mL/min/1.73 m^2^)** | **1498** | **80.7** | **<0.0001** | **-74.4** | **-5 mL/min/1.73 m^2^** | **1.15** | **1.12-1.19** | **<0.0001** |
| **eGFR (log-scale)** | **1498** | **76.0** | **<0.0001** | **-69.8** | **÷2** | **1.81** | **1.59-2.07** | **<0.0001** |
| **CKD stage** | **1498** | **76.1** | **<0.0001** | **-57.4** | **Stage 3b vs. stages 1, 2, and 3a** | **1.98** | **1.52-2.57** | **<0.0001** |
|  |  |  |  |  | **Stage 4 vs. stages 1, 2, and 3a** | **2.70** | **2.09-3.48** | **<0.0001** |
|  |  |  |  |  | **Stage 5 vs. stages 1, 2, and 3a** | **3.44** | **2.33-5.09** | **<0.0001** |
| **Age (years)** | **2192** | **447.6** | **<0.0001** | **-441.0** | **+10** | **1.89** | **1.77-2.02** | **0.0020** |
| **Sex** | **2192** | **9.7** | **0.0018** | **-3.1** | **Female vs. male** | **0.79** | **0.69-0.92** | **0.0020** |
| **Ethnic group** | 2189 | 18.7 | 0.0003 | 1.2 | **Asian vs. white** | **0.36** | **0.18-0.72** | **0.0039** |
|  |  |  |  |  | Black vs. white | 0.56 | 0.21-1.48 | 0.2409 |
|  |  |  |  |  | Other vs. white | 0.00 | 0.00->99 | 0.9844 |
| **Smoking status** | **2192** | **36.3** | **<0.0001** | **-23.0** | **Active smoker vs. non-smoker** | **1.39** | **1.09-1.76** | **0.0076** |
|  |  |  |  |  | **Former smoker vs. non-smoker** | **1.62** | **1.38-1.91** | **<0.0001** |
| **Diabetes mellitus** | **2192** | **50.0** | **<0.0001** | **-43.3** | **Yes vs. no** | **1.69** | **1.47-1.95** | **<0.0001** |
| **CVD** | **2192** | **168.2** | **<0.0001** | **-161.6** | **Yes vs. no** | **2.55** | **2.22-2.94** | **<0.0001** |
| **Cerebrovascular disease** | **2192** | **41.3** | **<0.0001** | **-34.7** | **Yes vs. no** | **2.06** | **1.69-2.53** | **<0.0001** |
| **PVD** | **2192** | **41.5** | **<0.0001** | **-34.9** | **Yes vs. no** | **1.82** | **1.54-2.17** | **<0.0001** |
| LVH | 2192 | 0.6 | 0.4386 | 6.1 | Yes vs. no | 1.31 | 0.68-2.54 | 0.4142 |
| **SBP (mmHg)** | **2161** | **15.6** | **<0.0001** | **-9.0** | **+10 mmHg** | **1.07** | **1.03-1.10** | **<0.0001** |
| **DBP (mmHg)** | **2152** | **40.1** | **<0.0001** | **-33.4** | **+10 mmHg** | **0.81** | **0.75-0.86** | **<0.0001** |
| **Pulse pressure (mmHg)** | **2152** | **66.3** | **<0.0001** | **-59.7** | **+10 mmHg** | **1.16** | **1.12-1.20** | **<0.0001** |
| Weight (kg) | 1825 | 1.4 | 0.2367 | 5.0 | +10 kg | 0.97 | 0.93-1.02 | 0.2341 |
| Height (m) | 2135 | 0.1 | 0.7518 | 6.6 | +10 cm | 0.99 | 0.92-1.06 | 0.7486 |
| BMI (kg/m^2^) | 1790 | 2.4 | 0.1213 | 4.0 | +5 kg/m^2^ | 0.95 | 0.88-1.02 | 0.1249 |
| **Hemoglobin (g/L)** | **1998** | **77.6** | **<0.0001** | **-70.9** | **+10 g/L** | **0.82** | **0.79-0.86** | **<0.0001** |
| **Hemoglobin <100 g/L** | **1998** | **30.9** | **<0.0001** | **-24.2** | **<100 vs. ≥100 g/L** | **2.22** | **1.72-2.87** | **<0.0001** |
| **Hemoglobin categories** | **1998** | **86.3** | **<0.0001** | **-59.7** | **<100 vs. ≥130 g/L** | **3.14** | **2.38-4.13** | **<0.0001** |
|  |  |  |  |  | **100-110 vs. ≥130 g/L** | **1.96** | **1.55-2.46** | **<0.0001** |
|  |  |  |  |  | **110-120 vs. ≥130 g/L** | **1.87** | **1.54-2.27** | **<0.0001** |
|  |  |  |  |  | **120-130 vs. ≥130 g/L** | **1.54** | **1.27-1.87** | **<0.0001** |
| **CRP (mg/L)** | **1710** | **50.7** | **<0.0001** | **-44.2** | **+5 mg/L** | **1.07** | **1.06-1.08** | **<0.0001** |
| **CRP (log-scale)** | **1710** | **109.7** | **<0.0001** | **-103.2** | **x2** | **1.27** | **1.21-1.32** | **<0.0001** |
| **CRP >5 mg/L** | **1710** | **82.0** | **<0.0001** | **-75.5** | **>5 vs. ≤5 mg/L** | **2.04** | **1.75-2.38** | **<0.0001** |
| **LDL cholesterol (calculated; mmol/L)** | **1517** | **34.4** | **<0.0001** | **-28.1** | **+1 mol/L** | **0.79** | **0.72-0.86** | **<0.0001** |
| **LDL cholesterol groups** | **2192** | **17.3** | **0.0002** | **-4.0** | **<3 vs. ≥3 mol/L** | **1.50** | **1.21-1.84** | **0.0002** |
|  |  |  |  |  | Missing vs. ≥3 mol/L | 1.22 | 0.97-1.53 | 0.0869 |
| Triglycerides (mmol/L) | 92 | 1.6 | 0.2059 | 2.3 | +1 mol/L | 0.88 | 0.69-1.11 | 0.2627 |
| Triglycerides groups | 2192 | 6.0 | 0.0498 | 7.3 | ≤2.3 vs. >2.3 mol/L | 1.33 | 0.68-2.61 | 0.4072 |
|  |  |  |  |  | Missing vs. >2.3 mol/L | 0.86 | 0.47-1.56 | 0.6229 |
| **Total cholesterol (mmol/L)** | **1861** | **48.1** | **<0.0001** | **-41.5** | **+1 mol/L** | **0.80** | **0.75-0.85** | **<0.0001** |
| **Total cholesterol >5 mmol/L** | **1861** | **25.8** | **<0.0001** | **-19.2** | **≥5 vs. <5 mol/L** | **0.66** | **0.56-0.78** | **<0.0001** |
| **Hematocrit (ratio)** | **1998** | **59.9** | **<0.0001** | **-53.2** | **+0.1 L/L** | **0.55** | **0.47-0.64** | **<0.0001** |
| **HDL cholesterol (mmol/L)** | **1521** | **5.2** | **0.0226** | **1.1** | **+0.5 mol/L** | **0.90** | **0.83-0.99** | **0.0252** |
| **Iron saturation (%)** | **1771** | **20.3** | **<0.0001** | **-13.8** | **+10%** | **0.85** | **0.79-0.91** | **<0.0001** |
| **Albumin (g/L)** | **2005** | **47.7** | **<0.0001** | **-41.1** | **-5 g/L** | **1.29** | **1.21-1.38** | **<0.0001** |
| **Phosphate (mmol/L)** | **1992** | **25.2** | **<0.0001** | **-18.6** | **+0.1 mmol/L** | **1.07** | **1.05-1.10** | **<0.0001** |
| **Corrected calcium (mmol/L)** | **2001** | **6.4** | **0.0114** | **0.2** | **+0.1 mmol/L** | **0.93** | **0.88-0.99** | **0.0126** |
| **PTH (ng/L)** | **1808** | **38.4** | **<0.0001** | **-31.8** | **+100 ng/L** | **1.31** | **1.21-1.42** | **<0.0001** |
| **PTH (log-scale)** | **1808** | **57.6** | **<0.0001** | **-51.0** | **x2** | **1.29** | **1.21-1.38** | **<0.0001** |
| uPCR (g/mol) | 1349 | 1.3 | 0.2542 | 4.8 | +100 g/mol | 1.03 | 0.98-1.09 | 0.2453 |
| **uPCR (log-scale)** | **1349** | **14.4** | **0.0001** | **-8.3** | **x2** | **1.10** | **1.05-1.16** | **0.0001** |
| **Sodium (mmol/L)** | **2007** | **11.4** | **0.0007** | **-4.8** | **+5 mmol/L** | **0.82** | **0.74-0.92** | **0.0006** |
| Potassium (mmol/L) | 1990 | 0.7 | 0.4028 | 6.0 | +5 mmol/L | 0.78 | 0.43-1.41 | 0.4084 |
| **Ferritin (µg/L)** | **1767** | **15.9** | **<0.0001** | **-9.4** | **+100 µg/L** | **1.06** | **1.04-1.09** | **<0.0001** |
| **Ferritin (log-scale)** | **1767** | **16.9** | **<0.0001** | **-10.4** | **x2** | **1.12** | **1.06-1.18** | **<0.0001** |
| **Ferritin groups** | 1767 | 22.2 | <0.0001 | -9.1 | 100 to <300 vs. <100 µg/L | 1.16 | 0.98-1.37 | 0.0885 |
|  |  |  |  |  | **≥300 vs. <100 µg/L** | **1.69** | **1.37-2.09** | **<0.0001** |
| HbA1c (mmol/mol) | 343 | 1.5 | 0.2207 | 3.6 | +10 mmol/mol | 1.05 | 0.97-1.14 | 0.2110 |
| **HbA1c groups** | 2192 | 26.6 | <0.0001 | 0.1 | 41 to <48 vs. ≤41 mmol/mol | 1.58 | 0.93-2.66 | 0.0879 |
|  |  |  |  |  | **48 to <75 vs. ≤41 mmol/mol** | **1.65** | **1.04-2.62** | **0.0343** |
|  |  |  |  |  | **>75 vs. ≤41 mmol/mol** | **1.88** | **1.08-3.27** | **0.0262** |
|  |  |  |  |  | Missing vs. ≤41 mmol/mol | 0.99 | 0.66-1.50 | 0.9790 |
| **ESA dose groups** | **2192** | **30.2** | **<0.0001** | **-16.9** | **ESA, <40 µg/2 weeks vs. no ESA** | **1.73** | **1.38-2.16** | **<0.0001** |
|  |  |  |  |  | **ESA, ≥40 µg/2 weeks vs. no ESA** | **1.78** | **1.32-2.41** | **0.0002** |
| **Renin-angiotensin blockade** | **2192** | **11.3** | **0.0008** | **-4.7** | **Yes vs. no** | **0.78** | **0.68-0.90** | **0.0007** |
| **Number of antihypertensives** | **2166** | **35.2** | **<0.0001** | **-28.5** | **+1** | **1.13** | **1.08-1.19** | **<0.0001** |
| **Lipid-lowering drugs** | **2192** | **27.8** | **<0.0001** | **-21.2** | **Yes vs. no** | **1.48** | **1.28-1.72** | **<0.0001** |
| **Anticoagulants** | **2192** | **101.8** | **<0.0001** | **-95.2** | **Yes vs. no** | **2.08** | **1.80-2.41** | **<0.0001** |

**Bold** and shaded rows indicate variables that are statistically significant (P<0.05).

^a^ Negative ΔBIC means that the variable improves the BIC.

BIC, Bayesian information criterion; BMI, body mass index; CKD, chronic kidney disease; CI, confidence interval; CRP, C-reactive protein; CVD, cardiovascular disease; DBP, diastolic blood pressure; eGFR, estimated glomerular filtration rate; ESA, erythropoiesis-stimulating agent; Hb, hemoglobin; HDL, high-density lipoprotein; LDL, low-density lipoprotein; LRT, likelihood ratio test; LVH, left ventricular hypertrophy; n, number of valid observations; PKD, polycystic kidney disease; PTH, parathyroid hormone; PVD, peripheral vascular disease; SBP, systolic blood pressure; SD, standard deviation; uPCR, urinary protein:creatinine ratio.

Table S5. Time to first myocardial infarction: univariate analysis results (proportional hazards approach)

| **Dependent variable** | **n** | **LRT** | **LRT  P value** | **ΔBIC^a^** | **Units used to build hazard ratio** | **Hazard ratio** | **95% CI** | **Wald P value** |
| --- | --- | --- | --- | --- | --- | --- | --- | --- |
| **Renal diagnosis** | 2192 | 23.2 | 0.0031 | 8.8 | Diabetic nephropathy vs. PKD | >99 | 0.00- >99 | 0.9917 |
|  |  |  |  |  | Hypertensive nephrosclerosis vs. PKD | >99 | 0.00- >99 | 0.9920 |
|  |  |  |  |  | Renovascular disease vs. PKD | >99 | 0.00- >99 | 0.9915 |
|  |  |  |  |  | Glomerulonephritis vs. PKD | >99 | 0.00- >99 | 0.9922 |
|  |  |  |  |  | Chronic pyelonephritis vs. PKD | >99 | 0.00- >99 | 0.9923 |
|  |  |  |  |  | Unknown vs. PKD | >99 | 0.00- >99 | 0.9920 |
|  |  |  |  |  | Others vs. PKD | >99 | 0.00- >99 | 0.9918 |
|  |  |  |  |  | **Missing vs. PKD** | **>99** | **0.00- >99** | **<0.0001** |
| **eGFR (mL/min/1.73 m^2^)** | **1498** | **8.0** | **0.0047** | **-4.5** | **-5 mL/min/1.73 m^2^** | **1.20** | **1.05-1.37** | **0.0085** |
| **eGFR (log-scale)** | **1498** | **5.9** | **0.0151** | **-2.5** | **÷2** | **1.88** | **1.14-3.11** | **0.0142** |
| **CKD stage** | **1498** | **8.6** | **0.0351** | **1.9** | **Stage 3b vs. stages 1, 2 and 3a** | **3.66** | **1.03-12.98** | **0.0448** |
|  |  |  |  |  | **Stage 4 vs. stages 1, 2 and 3a** | **4.48** | **1.29-15.52** | **0.0181** |
|  |  |  |  |  | **Stage 5 vs. stages 1, 2 and 3a** | **5.42** | **1.08-27.13** | **0.0397** |
| **Age (years)** | **2192** | **11.4** | **0.0007** | **-7.4** | **+10** | **1.41** | **1.14-1.76** | **0.0017** |
| Sex | 2192 | 2.5 | 0.1138 | 1.5 | Female vs. male | 0.63 | 0.35-1.13 | 0.1228 |
| **Ethnic group** | 2189 | 1.5 | 0.6823 | 10.5 | Asian vs. white | 1.38 | 0.34-5.68 | 0.6521 |
|  |  |  |  |  | **Black vs. white** | **0.00** | **0.1->99** | **<0.0001** |
|  |  |  |  |  | Other vs. white | 0.00 | 0.00->99 | 0.9929 |
| **Smoking status** | 2192 | 5.3 | 0.0707 | 2.7 | Active smoker vs. non-smoker | 1.73 | 0.68-4.39 | 0.2505 |
|  |  |  |  |  | **Former smoker vs. non-smoker** | **2.07** | **1.08-3.98** | **0.0292** |
| **Diabetes mellitus** | **2192** | **8.8** | **0.0030** | **-4.8** | **Yes vs. no** | **2.26** | **1.33-3.85** | **0.0025** |
| **CVD** | **2192** | **21.5** | **<0.0001** | **-17.5** | **Yes vs. no** | **3.55** | **2.06-6.13** | **<0.0001** |
| Cerebrovascular disease | 2192 | 1.5 | 0.2207 | 2.5 | Yes vs. no | 1.72 | 0.78-3.79 | 0.1826 |
| PVD | 2192 | 2.1 | 0.1473 | 1.9 | Yes vs. no | 1.67 | 0.86-3.23 | 0.1295 |
| LVH | 2192 | 1.0 | 0.3173 | 3.0 | Yes vs. no | 0.00 | 0.00->99 | 0.9923 |
| SBP (mmHg) | 2161 | 2.8 | 0.0943 | 1.2 | +10 mmHg | 1.11 | 0.98-1.26 | 0.0879 |
| DBP (mmHg) | 2152 | 0.1 | 0.7518 | 3.9 | +10 mmHg | 0.97 | 0.76-1.25 | 0.8342 |
| **Pulse pressure (mmHg)** | **2152** | **3.8** | **0.0513** | **0.2** | **+10 mmHg** | **1.14** | **1.00-1.31** | **0.0470** |
| Weight (kg) | 1825 | 0.3 | 0.5839 | 3.4 | +10 kg | 1.05 | 0.89-1.23 | 0.5813 |
| Height (m) | 2135 | 0.0 | 1.0000 | 3.8 | +10 cm | 1.04 | 0.79-1.36 | 0.7740 |
| BMI (kg/m^2^) | 1790 | 0.1 | 0.7518 | 3.6 | +5 kg/m^2^ | 1.04 | 0.81-1.35 | 0.7342 |
| Hemoglobin (g/L) | 1998 | 0.0 | 1.0000 | 4.0 | +10 g/L | 1.00 | 0.85-1.17 | 0.9878 |
| Hemoglobin <100 g/L | 1998 | 0.7 | 0.4028 | 3.3 | <100 vs. ≥100 g/L | 1.58 | 0.57-4.38 | 0.3830 |
| Hemoglobin categories | 1998 | 2.5 | 0.6446 | 13.5 | <100 vs. ≥130 g/L | 1.63 | 0.56-4.76 | 0.3708 |
|  |  |  |  |  | 100-110 vs. ≥130 g/L | 0.87 | 0.33-2.30 | 0.7785 |
|  |  |  |  |  | 110-120 vs. ≥130 g/L | 1.42 | 0.73-2.78 | 0.3048 |
|  |  |  |  |  | 120-130 vs. ≥130 g/L | 0.85 | 0.40-1.80 | 0.6754 |
| CRP (mg/L) | 1710 | 0.7 | 0.4028 | 3.1 | +5 mg/L | 1.04 | 0.96-1.11 | 0.3309 |
| CRP (log-scale) | 1710 | 4.0 | 0.0455 | -0.2 | x2 | 1.19 | 1.00-1.40 | 0.0452 |
| CRP >5 mg/L | 1710 | 2.4 | 0.1213 | 1.4 | >5 vs. ≤5 mg/L | 1.60 | 0.89-2.89 | 0.1174 |
| LDL cholesterol (calculated; mmol/L) | 1517 | 0.4 | 0.5271 | 3.3 | +1 mol/L | 0.91 | 0.69-1.22 | 0.5387 |
| LDL cholesterol groups | 2192 | 1.3 | 0.5220 | 6.7 | <3 vs. ≥3 mol/L | 1.42 | 0.66-3.09 | 0.3722 |
|  |  |  |  |  | Missing vs. ≥3 mol/L | 1.06 | 0.45-2.51 | 0.8889 |
| Triglycerides (mmol/L) | 92 | 1.7 | 0.1923 | 0.2 | +1 mol/L | 0.56 | 0.18-1.68 | 0.2928 |
| Triglycerides groups | 2192 | 6.7 | 0.0351 | 1.3 | ≤2.3 vs. >2.3 mol/L | 2.16 | 0.26-17.99 | 0.4749 |
|  |  |  |  |  | Missing vs. >2.3 mol/L | 0.58 | 0.08-4.23 | 0.5941 |
| Total cholesterol (mmol/L) | 1861 | 1.7 | 0.1923 | 2.3 | +1 mol/L | 0.86 | 0.67-1.09 | 0.2071 |
| Total cholesterol >5 mmol/L | 1861 | 0.7 | 0.4028 | 3.2 | ≥5 vs. <5 mol/L | 0.78 | 0.43-1.41 | 0.4056 |
| Hematocrit (ratio) | 1998 | 0.0 | 1.0000 | 4.0 | +0.1 L/L | 0.96 | 0.54-1.68 | 0.8737 |
| HDL cholesterol (mmol/L) | 1521 | 2.0 | 0.1573 | 1.7 | +0.5 mol/L | 0.78 | 0.55-1.12 | 0.1734 |
| Iron saturation (%) | 1771 | 0.7 | 0.4028 | 3.2 | +10% | 1.10 | 0.87-1.41 | 0.4252 |
| Albumin (g/L) | 2005 | 2.7 | 0.1003 | 1.2 | -5 g/L | 1.27 | 0.98-1.64 | 0.0725 |
| Phosphate (mmol/L) | 1992 | 0.0 | 1.0000 | 4.0 | +0.1 mmol/L | 0.99 | 0.89-1.11 | 0.8782 |
| Corrected calcium (mmol/L) | 2001 | 0.8 | 0.3711 | 3.2 | +0.1 mmol/L | 0.91 | 0.75-1.12 | 0.3783 |
| PTH (ng/L) | 1808 | 0.1 | 0.7518 | 3.8 | +100 ng/L | 0.95 | 0.63-1.43 | 0.8021 |
| PTH (log-scale) | 1808 | 0.2 | 0.6547 | 3.7 | x2 | 0.95 | 0.74-1.22 | 0.6643 |
| uPCR (g/mol) | 1349 | 0.1 | 0.7518 | 3.3 | +100 g/mol | 0.98 | 0.76-1.26 | 0.8486 |
| uPCR (log-scale) | 1349 | 0.7 | 0.4028 | 2.7 | x2 | 1.08 | 0.89-1.31 | 0.4249 |
| Sodium (mmol/L) | 2007 | 0.6 | 0.4386 | 3.4 | +5 mmol/L | 0.85 | 0.56-1.29 | 0.4412 |
| Potassium (mmol/L) | 1990 | 0.1 | 0.7518 | 3.9 | +5 mmol/L | 1.30 | 0.14-11.75 | 0.8171 |
| Ferritin (µg/L) | 1767 | 0.9 | 0.3428 | 2.9 | +100 µg/L | 1.06 | 0.95-1.17 | 0.2941 |
| Ferritin (log-scale) | 1767 | 0.2 | 0.6547 | 3.6 | x2 | 1.04 | 0.85-1.28 | 0.7135 |
| Ferritin groups | 1767 | 2.8 | 0.2466 | 4.9 | 100 to <300 vs. <100 µg/L | 0.70 | 0.35-1.39 | 0.3081 |
|  |  |  |  |  | ≥300 vs. <100 µg/L | 1.43 | 0.66-3.11 | 0.3693 |
| HbA1c (mmol/mol) | 343 | 0.0 | 1.0000 | 2.6 | +10 mmol/mol | 1.04 | 0.80-1.34 | 0.7882 |
| HbA1c groups | 2192 | 12.3 | 0.0153 | 3.7 | 41 to <48 vs. ≤41 mmol/mol | 0.58 | 0.05-6.35 | 0.6518 |
|  |  |  |  |  | 48 to <75 vs. ≤41 mmol/mol | 3.02 | 0.67-13.64 | 0.1506 |
|  |  |  |  |  | >75 vs. ≤41 mmol/mol | 0.79 | 0.07-8.67 | 0.8434 |
|  |  |  |  |  | Missing vs. ≤41 mmol/mol | 0.78 | 0.19-3.23 | 0.7321 |
| ESA dose groups | 2192 | 0.2 | 0.9048 | 7.8 | ESA, <40 µg/2 weeks vs. no ESA | 1.27 | 0.51-3.21 | 0.6063 |
|  |  |  |  |  | ESA, ≥40 µg/2 weeks vs. no ESA | 0.98 | 0.24-4.04 | 0.9775 |
| **Renin-angiotensin blockade** | **2192** | **6.8** | **0.0091** | **-2.8** | **Yes vs. no** | **0.49** | **0.29-0.84** | **0.0089** |
| Number of antihypertensives | 2166 | 0.2 | 0.6547 | 3.7 | +1 | 0.99 | 0.82-1.20 | 0.9160 |
| **Lipid-lowering drugs** | **2192** | **8.8** | **0.0030** | **-4.8** | **Yes vs. no** | **2.46** | **1.30-4.67** | **0.0059** |
| **Anticoagulants** | **2192** | **11.9** | **0.0006** | **-8.0** | **Yes vs. no** | **2.65** | **1.48-4.75** | **0.0010** |

**Bold** and shaded rows indicate variables that are statistically significant (P<0.05).

^a^ Negative ΔBIC means that the variable improves the BIC.

BIC, Bayesian information criterion; BMI, body mass index; CKD, chronic kidney disease; CI, confidence interval; CRP, C-reactive protein; CVD, cardiovascular disease; DBP, diastolic blood pressure; eGFR, estimated glomerular filtration rate; ESA, erythropoiesis-stimulating agent; Hb, hemoglobin; HDL, high-density lipoprotein; LDL, low-density lipoprotein; LRT, likelihood ratio test; LVH, left ventricular hypertrophy; n, number of valid observations; PKD, polycystic kidney disease; PTH, parathyroid hormone; PVD, peripheral vascular disease; SBP, systolic blood pressure; SD, standard deviation; uPCR, urinary protein:creatinine ratio.

Table S6. Time to renal replacement therapy in patients with non‒dialysis-dependent chronic kidney disease: final joint model

| **Variable** | **Units used to build hazard ratio** | **Hazard ratio  (95% CI)** | **Wald P value** |
| --- | --- | --- | --- |
| **Renal diagnosis** | **Diabetic nephropathy vs. PKD** | **0.48 (0.26-0.88)** | **0.018** |
|  | **Hypertensive nephrosclerosis vs. PKD** | **0.36 (0.21-0.60)** | **<0.001** |
|  | Renovascular disease vs. PKD | 0.54 (0.27-1.09) | 0.085 |
|  | **Glomerulonephritis vs. PKD** | **0.30 (0.18-0.51)** | **<0.001** |
|  | **Chronic pyelonephritis vs. PKD** | **0.47 (0.25-0.89)** | **0.021** |
|  | **Unknown vs. PKD** | **0.50 (0.29-0.85)** | **0.011** |
|  | **Others vs. PKD** | **0.51 (0.31-0.84)** | **0.008** |
|  | **Missing vs. PKD** | **4.12 (1.95-8.70)** | **<0.001** |
| **eGFR (mL/min/1.73 m^2^)** | **÷2** | **3.40 (2.65-4.35)** | **<0.001** |
| **eGFR (log-scale)** | **÷2 per year** | **45.89 (23.33-90.27)** | **<0.001** |
| Age (years) | +10 years | 0.95 (0.87-1.05) | 0.347 |
| **Sex** | **Female vs. male** | **0.53 (0.40-0.70)** | **<0.001** |
| Diabetes mellitus | Yes vs. no | 1.10 (0.69-1.75) | 0.686 |
| CVD | Yes vs. no | 1.21 (0.92-1.59) | 0.177 |
| **SBP (mmHg)** | **+10 mmHg** | **1.08 (1.01-1.14)** | **0.014** |
| Hemoglobin (g/L) | +10 g/L | 0.94 (0.85-1.03) | 0.188 |
| Albumin (g/L) | -5 g/L | 1.02 (0.86-1.21) | 0.802 |
| uPCR (log-scale) | x2 | 1.06 (0.97-1.16) | 0.203 |
| **Anticoagulants** | **Yes vs. no** | **1.23 (1.12-1.34)** | **<0.001** |

**Bold** rows indicate variables that are statistically significant (P<0.05).

CVD, cardiovascular disease; eGFR, estimated glomerular filtration rate; PKD, polycystic kidney disease; SBP, systolic blood pressure; uPCR, urinary protein:creatinine ratio.

Table S7. Post hoc analysis: Time to first MACE^a^: univariate analysis results (proportional hazards approach)

| **Dependent variable** | **n** | **LRT** | **LRT P value** | **ΔBIC^b^** | **Units used to build hazard ratio** | **Hazard ratio** | **95% CI** | **Wald P value** |
| --- | --- | --- | --- | --- | --- | --- | --- | --- |
| **Renal diagnosis** | **2192** | **241.9** | **<.0001** | **-188.6** | **Diabetic nephropathy vs. PKD** | **6.22** | **3.18-12.14** | **<0.0001** |
|  |  |  |  |  | **Hypertensive nephrosclerosis vs. PKD** | **4.73** | **2.40-9.32** | **<0.0001** |
|  |  |  |  |  | **Renovascular disease vs. PKD** | **9.45** | **4.79-18.62** | **<0.0001** |
|  |  |  |  |  | **Glomerulonephritis vs. PKD** | **2.49** | **1.24-4.99** | **0.0100** |
|  |  |  |  |  | Chronic pyelonephritis vs. PKD | 1.88 | 0.89-3.97 | 0.0981 |
|  |  |  |  |  | **Unknown vs. PKD** | **4.93** | **2.51-9.70** | **<.0001** |
|  |  |  |  |  | **Others vs. PKD** | **3.09** | **1.57-6.10** | **0.0011** |
|  |  |  |  |  | Missing vs. PKD | 0.54 | 0.20-1.46 | 0.2285 |
| **eGFR (mL/min/1.73 m^2^)** | **1498** | **84.2** | **<.0001** | **-78.0** | **-5 mL/min/1.73 m^2^** | **1.16** | **1.12-1.20** | **<0.0001** |
| **eGFR (log-scale)** | **1498** | **79.6** | **<.0001** | **-73.4** | **÷2** | **1.84** | **1.61-2.11** | **<0.0001** |
| **CKD stage** | **1498** | **79.9** | **<.0001** | **-61.3** | **Stage 3b vs. stages 1, 2, and 3a** | **1.99** | **1.52-2.60** | **<0.0001** |
|  |  |  |  |  | **Stage 4 vs. stages 1, 2, and 3a** | **2.78** | **2.15-3.60** | **<0.0001** |
|  |  |  |  |  | **Stage 5 vs. stages 1, 2, and 3a** | **3.58** | **2.42-5.30** | **<0.0001** |
| **Age (years)** | **2192** | **453.2** | **<.0001** | **-446.5** | **+10** | **1.91** | **1.79-2.05** | **<0.0001** |
| **Sex** | **2192** | **10.8** | **0.0010** | **-4.1** | **Female vs. male** | **0.78** | **0.67-0.91** | **0.0012** |
| **Ethnic group** | **2189** | **17.9** | **0.0005** | **2.1** | **Asian vs. white** | **0.37** | **0.18-0.74** | **0.0048** |
|  |  |  |  |  | Black vs. white | 0.57 | 0.21-1.53 | 0.2632 |
|  |  |  |  |  | Other vs. white | 0.00 | 0.00->99 | 0.9668 |
| **Smoking status** | **2192** | **36.0** | **<.0001** | **-22.7** | **Active vs. non-smoker** | **1.42** | **1.12-1.81** | **0.0041** |
|  |  |  |  |  | **Former vs. non-smoker** | **1.62** | **1.38-1.91** | **<0.0001** |
| **Diabetes mellitus** | **2192** | **49.0** | **<.0001** | **-42.4** | **Yes vs. no** | **1.69** | **1.46-1.95** | **<0.0001** |
| **CVD** | **2192** | **152.5** | **<.0001** | **-145.8** | **Yes vs. no** | **2.46** | **2.13-2.83** | **<0.0001** |
| **Cerebrovascular disease** | **2192** | **37.0** | **<.0001** | **-30.3** | **Yes vs. no** | **2.00** | **1.63-2.45** | **<0.0001** |
| **PVD** | **2192** | **36.6** | **<.0001** | **-30.0** | **Yes vs. no** | **1.77** | **1.48-2.10** | **<0.0001** |
| LVH | 2192 | 0.7 | 0.4028 | 6.0 | Yes vs. no | 1.34 | 0.70-2.59 | 0.3782 |
| **SBP (mmHg)** | **2161** | **17.0** | **<.0001** | **-10.4** | **+10 mmHg** | **1.07** | **1.04-1.11** | **<0.0001** |
| **DBP (mmHg)** | **2152** | **38.0** | **<.0001** | **-31.3** | **+10 mmHg** | **0.81** | **0.76-0.87** | **<0.0001** |
| **Pulse pressure (mmHg)** | **2152** | **67.8** | **<.0001** | **-61.1** | **+10 mmHg** | **1.16** | **1.12-1.21** | **<0.0001** |
| Weight (kg) | 1825 | 1.3 | 0.2542 | 5.1 | +10 kg | 0.97 | 0.93-1.02 | 0.2530 |
| Height (m) | 2135 | 0.0 | 1.0000 | 6.6 | +10 cm | 1.00 | 0.93-1.07 | 0.9681 |
| BMI (kg/m^2^) | 1790 | 2.2 | 0.1380 | 4.2 | +5 kg/m^2^ | 0.95 | 0.89-1.02 | 0.1403 |
| **Hemoglobin (g/L)** | **1998** | **76.3** | **<.0001** | **-69.7** | **+10 g/L** | **0.82** | **0.79-0.86** | **<0.0001** |
| **Hemoglobin <100 g/L** | **1998** | **32.5** | **<.0001** | **-25.9** | **<100 vs. ≥100 g/L** | **2.28** | **0.79-0.86** | **<0.0001** |
| **Hemoglobin categories** | **1998** | **86.0** | **<.0001** | **-59.5** | **<100 vs. ≥130 g/L** | **3.21** | **2.43-4.22** | **<0.0001** |
|  |  |  |  |  | **100-110 vs. ≥130 g/L** | **1.95** | **1.54-2.45** | **<0.0001** |
|  |  |  |  |  | **110-120 vs. ≥130 g/L** | **1.85** | **1.52-2.25** | **<0.0001** |
|  |  |  |  |  | **120-130 vs. ≥130 g/L** | **1.55** | **1.28-1.89** | **<0.0001** |
| **CRP (mg/L)** | **1710** | **51.5** | **<.0001** | **-45.0** | **+5 mg/L** | **1.07** | **1.06-1.09** | **<0.0001** |
| **CRP (log-scale)** | **1710** | **109.1** | **<.0001** | **-102.6** | **x2** | **1.27** | **1.21-1.33** | **<0.0001** |
| **CRP >5 mg/L** | **1710** | **82.9** | **<.0001** | **-76.4** | **>5 vs. ≤5 mg/L** | **2.06** | **1.76-2.40** | **<0.0001** |
| **LDL cholesterol (calculated; mmol/L)** | **1517** | **32.6** | **<.0001** | **-26.2** | **+1 mol/L** | **0.79** | **0.73-0.86** | **<0.0001** |
| **LDL cholesterol groups** | **2192** | **16.9** | **0.0002** | **-3.6** | **<3 mmol/L vs. ≥3 mmol/L** | **1.47** | **1.19-1.81** | **0.0003** |
|  |  |  |  |  | Missing vs. ≥3 mmol/L | 1.17 | 0.93-1.47 | 0.1707 |
| Triglycerides (mmol/L) | 92 | 1.4 | 0.2367 | 2.4 | +1 mmol/L | 0.88 | 0.70-1.11 | 0.2844 |
| Triglycerides groups | 2192 | 5.5 | 0.0639 | 7.9 | ≤2.3 mmol/L vs. >2.3 mmol/L | 1.28 | 0.65-2.51 | 0.4803 |
|  |  |  |  |  | Missing vs. >2.3 mmol/L | 0.84 | 0.46-1.53 | 0.5740 |
| **Total cholesterol (mmol/L)** | **1861** | **44.7** | **<.0001** | **-38.1** | **+1 mmol/L** | **0.80** | **0.75-0.86** | **<0.0001** |
| **Total cholesterol >5 mmol/L** | **1861** | **22.7** | **<.0001** | **-16.1** | **≥5 vs. <5 mmol/L** | **0.68** | **0.58-0.80** | **<0.0001** |
| **Hematocrit (ratio)** | **1998** | **58.2** | **<.0001** | **-51.6** | **+0.1 L/L** | **0.55** | **0.47-0.64** | **<0.0001** |
| **HDL cholesterol (mmol/L)** | **1521** | **5.4** | **0.0201** | **0.9** | **+0.5 mmol/L** | **0.90** | **0.82-0.99** | **0.0229** |
| **Iron saturation (%)** | **1771** | **19.0** | **<.0001** | **-12.5** | **+10 %** | **0.85** | **0.79-0.92** | **<0.0001** |
| **Albumin (g/L)** | **2005** | **45.9** | **<.0001** | **-39.2** | **-5 g/L** | **1.29** | **1.21-1.38** | **<0.0001** |
| **Phosphate (mmol/L)** | **1992** | **25.7** | **<.0001** | **-19.0** | **+0.1 mmol/L** | **1.08** | **1.05-1.11** | **<0.0001** |
| **Corrected calcium (mmol/L)** | **2001** | **7.1** | **0.0077** | **-0.4** | **+0.1 mmol/L** | **0.93** | **0.88-0.98** | **0.0090** |
| **PTH (ng/L)** | **1808** | **39.8** | **<.0001** | **-33.2** | **+100 ng/L** | **1.32** | **1.22-1.43** | **<0.0001** |
| **PTH (log-scale)** | **1808** | **58.3** | **<.0001** | **-51.8** | **x2** | **1.29** | **1.21-1.38** | **<0.0001** |
| uPCR (g/mol) | 1349 | 1.3 | 0.2542 | 4.7 | +100 g/mol | 1.03 | 0.98-1.09 | 0.2361 |
| **uPCR (log-scale)** | **1349** | **14.0** | **0.0002** | **-7.9** | **x2** | **1.10** | **1.05-1.16** | **0.0002** |
| **Sodium (mmol/L)** | **2007** | **11.0** | **0.0009** | **-4.4** | **+5 mmol/L** | **0.82** | **0.74-0.92** | **0.0007** |
| Potassium (mmol/L) | 1990 | 0.6 | 0.4386 | 6.0 | +5 mmol/L | 0.78 | 0.43-1.43 | 0.4303 |
| **Ferritin (µg/L)** | **1767** | **17.8** | **<.0001** | **-11.3** | **+100 µg/L** | **1.07** | **1.04-1.09** | **<0.0001** |
| **Ferritin (log-scale)** | **1767** | **19.7** | **<.0001** | **-13.2** | **x2** | **1.13** | **1.07-1.19** | **<0.0001** |
| **Ferritin groups** | **1767** | **25.2** | **<.0001** | **-12.1** | 100 to <300 µg/L vs. <100 µg/L | 1.18 | 0.99-1.39 | 0.0578 |
|  |  |  |  |  | **≥300 µg/L vs. <100 µg/L** | **1.75** | **1.42-2.16** | **<0.0001** |
| HbA1c (mmol/mol) | 343 | 0.8 | 0.3711 | 4.2 | +10 mmol/mol | 1.04 | 0.96-1.13 | 0.3466 |
| **HbA1c groups** | **2192** | **24.4** | **<.0001** | **2.2** | 41 to <48 vs. ≤41 mmol/mol | 1.62 | 0.95-2.76 | 0.0768 |
|  |  |  |  |  | **48 to <75 vs. ≤41 mmol/mol** | **1.74** | **1.09-2.78** | **0.0212** |
|  |  |  |  |  | >75 vs. ≤41 mmol/mol | 1.71 | 0.97-3.04 | 0.0648 |
|  |  |  |  |  | Missing vs. ≤41 mmol/mol | 1.03 | 0.68-1.56 | 0.8893 |
| **ESA dose groups** | **2192** | **33.3** | **<.0001** | **-20.0** | **ESA, <40 µg/2 weeks vs. no ESA** | **1.78** | **1.42-2.23** | **<0.0001** |
|  |  |  |  |  | **ESA, ≥40 µg/2 weeks vs. no ESA** | **1.84** | **1.36-2.48** | **<0.0001** |
| **Renin-angiotensin blockade** | **2192** | **10.2** | **0.0014** | **-3.5** | **Yes vs. no** | **0.79** | **0.69-0.91** | **0.0013** |
| **Number of antihypertensives** | **2166** | **33.5** | **<.0001** | **-26.8** | **+1** | **1.13** | **1.08-1.19** | **<0.0001** |
| **Lipid-lowering drugs** | **2192** | **27.1** | **<.0001** | **-20.5** | **Yes vs. no** | **1.48** | **1.27-1.72** | **<0.0001** |
| **Anticoagulants** | **2192** | **97.4** | **<.0001** | **-90.8** | **Yes vs. no** | **2.06** | **1.78-2.38** | **<0.0001** |

**Bold** and shaded rows indicate variables that are statistically significant (P<0.05).

^a^ Composite of non-fatal MI, non-fatal stroke, or all-cause mortality.

^b^ Negative ΔBIC means that the variable improves the BIC.

BIC, Bayesian information criterion; BMI, body mass index; CKD, chronic kidney disease; CI, confidence interval; CRP, C-reactive protein; CVD, cardiovascular disease; DBP, diastolic blood pressure; eGFR, estimated glomerular filtration rate; ESA, erythropoiesis-stimulating agent; Hb, hemoglobin; HDL, high-density lipoprotein; LDL, low-density lipoprotein; LRT, likelihood ratio test; LVH, left ventricular hypertrophy; n, number of valid observations; PKD, polycystic kidney disease; PTH, parathyroid hormone; PVD, peripheral vascular disease; SBP, systolic blood pressure; SD, standard deviation; uPCR, urinary protein:creatinine ratio.

Table S8. Post hoc analysis: Time to first MACE^a^: Final joint model

| **Dependent variable** | **Units used to build hazard ratio** | **Hazard ratio** | **95% CI** | **Wald P value** |
| --- | --- | --- | --- | --- |
| **Estimated eGFR at time t^b^** | **-5 mL/min/1.73 m^2^** | **1.04** | **1.00-1.08** | **0.0197** |
| Estimated eGFR slope | -5 mL/min/1.73 m^2^ per year | 1.03 | 0.80-1.35 | 0.7870 |
| **Age (years)** | **+10 years** | **1.92** | **1.78-2.07** | **<0.0001** |
| **Sex** | **Female vs. male** | **0.81** | **0.69-0.95** | **0.0088** |
| **Diabetes mellitus** | **Yes vs. no** | **1.23** | **1.06-1.43** | **0.0062** |
| **CVD** | **Yes vs. no** | **1.42** | **1.22-1.66** | **<0.0001** |
| **Cerebrovascular disease** | **Yes vs. no** | **1.30** | **1.05-1.61** | **0.0158** |
| **PVD** | **Yes vs. no** | **1.48** | **1.23-1.78** | **<0.0001** |
| **Hemoglobin (g/L)** | **+10 g/L** | **0.94** | **0.90-0.99** | **0.0288** |
| **Albumin (g/L)** | **-5 g/L** | **1.20** | **1.09-1.32** | **0.0002** |
| **Phosphate (mml/L)** | **+0.1 mmol/L** | **1.05** | **1.02-1.08** | **0.0034** |
| PTH (log-scale) | x2 | 1.07 | 0.99-1.15 | 0.1016 |
| uPCR (log-scale) | x2 | 1.07 | 1.02-1.13 | 0.0092 |

**Bold** and shaded rows indicate variables that are statistically significant (P≤0.05).

^a^ Composite of non-fatal MI, non-fatal stroke, or all-cause mortality.

^b^ Time t = arbitrary point in time between baseline and end of follow-up.

CI, confidence interval; CVD, cardiovascular disease; eGFR, estimated glomerular filtration rate; MI, myocardial infarction; PVD, peripheral vascular disease; uPCR, urinary protein:creatinine ratio.

Table S9. Subgroup analysis results for MACE: Significant variables in final joint model^a^

| **Variable** | | **Total cohort** | | **CRP** | | | | **Ferritin** | | | | | |
| --- | --- | --- | --- | --- | --- | --- | --- | --- | --- | --- | --- | --- | --- |
|  |  |  |  | **≤5 mg/L** | | **>5 mg/L** | | **<100 µg/L** | | **100 to <300 µg/L** | | **≥300 µg/L** | |
| Number of patients | | 2192 | | 1082 | | 628 | | 750 | | 727 | | 290 | |
| Number of events | | 780 | | 335 | | 321 | | 283 | | 267 | | 126 | |
| Time to event, mean (SD), y | | 4.3 (3.1) | | 4.6 (3.2) | | 3.7 (2.8) | | 4.8 (3.4) | | 4.2 (2.8) | | 3.2 (2.7) | |
| **Variable** | **Units used to build HR** | **HR** | **P value** | **HR** | **P value** | **HR** | **P value** | **HR** | **P value** | **HR** | **P value** | **HR** | **P value** |
| Estimated eGFR at time t | -5 mL/min/1.73 m^2^ | **1.08** | **0.02** | 1.04 | 0.068 | **1.05** | **0.033** | **1.07** | **0.005** | 1.02 | 0.43 | 1.01 | 0.84 |
| Estimated eGFR slope | -5 mL/min/1.73 m^2^ per year | 1.07 | 0.79 | 0.88 | 0.56 | 1.04 | 0.8 | 1.31 | 0.32 | 0.75 | 0.15 | 1.40 | 0.31 |
| Age (years) | +10 years | **1.92** | **<0.0001** | **2.1** | **<0.001** | **1.78** | **<0.001** | **1.88** | **<0.001** | **2.11** | **<0.001** | **1.78** | **<0.001** |
| Sex | Female vs. male | **0.81** | **0.0088** |  |  | **0.7** | **0.004** | **0.75** | **0.027** |  |  |  |  |
| Smoking status | Active vs. non |  |  | **1.93** | **<0.001** |  |  |  |  |  |  |  |  |
|  | Former vs. no |  |  | 0.99 | 0.91 |  |  |  |  |  |  |  |  |
| Diabetes mellitus | Yes vs. no | **1.23** | **0.0062** | 1.15 | 0.21 | **1.36** | **0.012** | 1.27 | 0.069 | 1.24 | 0.1 | 1.02 | 0.92 |
| CVD | Yes vs. no | **1.42** | **<0.0001** | **1.28** | **0.043** | **1.38** | **0.009** | **1.42** | **0.008** | **1.63** | **<0.001** | 1.18 | 0.4 |
| Cerebrovascular disease | Yes vs. no | **1.30** | **0.0158** |  |  | **1.58** | **0.009** | **1.59** | **0.009** |  |  | **2.39** | **0.004** |
| PVD | Yes vs. no | **1.48** | **<0.0001** |  |  |  |  | **1.89** | **<0.0001** |  |  |  |  |
| Hemoglobin (g/L) | +10 g/L | **0.94** | **0.0288** | 1.00 | 0.95 | 0.93 | 0.075 | 0.94 | 0.15 | **0.87** | **0.002** | 0.97 | 0.58 |
| CRP (log-scale) | x2 |  |  |  |  |  |  | **1.09** | **0.038** | **1.19** | **<0.001** |  |  |
| Albumin (g/L) | -5 g/L | **1.20** | **0.0002** | 1.08 | 0.31 | **1.24** | **0.006** | **1.26** | **0.008** | 1.03 | 0.76 | 1.18 | 0.12 |
| Phosphate (mml/L) | +0.1 mmol/L | **1.05** | **0.0034** | **1.08** | **0.005** |  |  |  |  |  |  |  |  |
| uPCR (log-scale) | x2 | **1.07** | **0.009** | **1.1** | **0.034** | 1.06 | 0.16 | 1.05 | 0.30 | **1.19** | **0.001** | 1.04 | 0.52 |
| PTH (log-scale) | x2 | 1.07 | 0.102 |  |  |  |  |  |  |  |  |  |  |
| Sodium | +5 mmol/L |  |  |  |  |  |  |  |  | **0.81** | **0.026** |  |  |
| ESA dose groups | <40 µg/2 weeks vs. none |  |  |  |  |  |  |  |  |  |  | 1.42 | 0.13 |
|  | ≥40 µg/2 weeks vs. none |  |  |  |  |  |  |  |  |  |  | 1.19 | 0.68 |
| Renin-angiotensin blockade | Yes vs. no |  |  |  |  |  |  | **0.68** | **0.002** |  |  |  |  |
| Number of antihypertensives | +1 |  |  |  |  | 0.94 | 0.2 |  |  |  |  |  |  |
| Anticoagulants | Yes vs. no |  |  | **1.39** | **0.013** |  |  |  |  |  |  |  |  |
| Log-ferritin by log-CRP interaction | x2 and x2 | Not performed | |  |  | **1.02** | **0.004** |  |  |  |  | **1.02** | **0.001** |

**Bold** indicates variables that are statistically significant (P<0.05).

^a^ Blank cells indicate results were not significant.

CRP, C-reactive protein; CVD, cardiovascular disease; eGFR, estimated glomerular filtration rate; HR, hazard ratio; MACE, Major adverse cardiovascular event; PVD, peripheral vascular disease; uPCR, urinary protein:creatinine ratio.

Table S10. Subgroup analysis results for CV-MACE: Significant variables in final joint model^a^

| **Variable** | | **Total cohort** | | **CRP** | | | | **Ferritin** | | | | | |
| --- | --- | --- | --- | --- | --- | --- | --- | --- | --- | --- | --- | --- | --- |
|  |  |  |  | **≤5 mg/L** | | **>5 mg/L** | | **<100 µg/L** | | **100 to <300 µg/L** | | **≥300 µg/L** | |
| Number of patients | | 2192 | | 1082 | | 628 | | 750 | | 727 | | 290 | |
| Number of events | | 422 | | 177 | | 174 | | 158 | | 140 | | 68 | |
| Time to event, mean (SD), y | | 4.5 (3.4) | | 4.7 (3.4) | | 3.9 (3.1) | | 5.1 (3.7) | | 4.3 (3.0) | | 3.3 (2.8) | |
| **Variable** | **Units used to build HR** | **HR** | **P value** | **HR** | **P value** | **HR** | **P value** | **HR** | **P value** | **HR** | **P value** | **HR** | **P value** |
| Estimated eGFR at time t | -5 mL/min/1.73 m^2^ | **1.06** | **0.012** | 1.06 | 0.06 | 1.02 | 0.43 | 1.05 | 0.12 | 1.03 | 0.4 | 1.05 | 0.42 |
| Estimated eGFR slope | -5 mL/min/1.73 m^2^ per year | 1.06 | 0.88 | 0.92 | 0.73 | 0.82 | 0.39 | 1.30 | 0.46 | 1.11 | 0.71 | 0.57 | 0.092 |
| Age (years) | +10 years | **1.6** | **<0.0001** | **2.00** | **<0.001** | **1.51** | **<0.001** | **1.71** | **<0.001** | **2.09** | **<0.001** | **1.52** | **0.002** |
| Diabetes mellitus | Yes vs. no | **1.39** | **0.0016** | 1.19 | 0.28 | **1.58** | **0.004** | **1.57** | **0.009** | 1.21 | 0.31 | 1.07 | 0.79 |
| CVD | Yes vs. no | **1.71** | **<0.0001** | **<0.0001** | **0.012** | **1.7** | **0.002** | **1.64** | **0.004** | **2.09** | **<0.001** | **1.92** | **0.021** |
| Cerebrovascular disease | Yes vs. no |  |  |  |  | **1.75** | **0.01** |  |  |  |  | **3.42** | **0.002** |
| PVD | Yes vs. no |  |  |  |  | **1.68** | **0.004** | **1.88** | **0.002** |  |  |  |  |
| Hemoglobin (g/L) | +10 g/L | 1.02 | 0.63 | 1.02 | 0.67 | 0.98 | 0.73 | 1.06 | 0.28 | 0.97 | 0.65 | 0.91 | 0.27 |
| Albumin (g/L) | -5 g/L | **1.2** | **0.006** | 1.17 | 0.14 | **1.38** | **0.002** | **1.44** | **0.001** | 0.95 | 0.68 | 1.2 | 0.18 |
| uPCR (log-scale) | x2 | 1.06 | 0.09 | 1.11 | 0.087 | 1.04 | 0.48 | 1.04 | 0.51 | 1.13 | 0.076 | 1.13 | 0.16 |
| Sodium | +5 mmol/L |  |  |  |  |  |  |  |  | **0.71** | **0.008** |  |  |
| ESA dose groups | <40 µg/2 weeks vs. none | **1.61** | **0.002** |  |  | **2.12** | **<0.001** |  |  |  |  | **2.99** | **<0.001** |
|  | ≥40 µg/2 weeks vs. none | **1.59** | **0.044** |  |  | 1.05 | 0.9 |  |  |  |  | 0.74 | 0.69 |
| Renin-angiotensin blockade | Yes vs. no |  |  |  |  |  |  | **0.58** | **0.001** |  |  |  |  |
| Number of antihypertensives | +1 | 1.06 | 0.1005 |  |  |  |  |  |  | **1.23** | **0.001** | 0.95 | 0.57 |
| Log-ferritin by log-CRP interaction | x2 and x2 | **1.02** | **0.001** |  |  |  |  |  |  |  |  |  |  |
| CRP (log-scale) | x2 |  |  |  |  |  |  |  |  | **1.24** | **<0.001** |  |  |

**Bold** indicates variables that are statistically significant (P<0.05).

^a^ Blank cells indicate results were not significant.

CRP, C-reactive protein; CV-MACE, cardiovascular major adverse cardiovascular event; CVD, cardiovascular disease; eGFR, estimated glomerular filtration rate; HR, hazard ratio; PVD, peripheral vascular disease; uPCR, urinary protein:creatinine ratio.

Table S11. Time to first CV-MACE: Final joint model, sensitivity analysis without forcing the retention of clinically important variables

| **Dependent variable** | **Change modelled** | **Hazard ratio** | **95% CI** | **Wald P value** |
| --- | --- | --- | --- | --- |
| Estimated eGFR at time t | -5 mL/min/1.73 m^2^ | 1.04 | 0.99-1.10 | 0.1252 |
| Estimated eGFR slope | -5 mL/min/1.73 m^2^ per year | 1.34 | 0.88-2.03 | 0.1692 |
| **Age, years** | **+10 years** | **1.39** | **1.21-1.60** | **<0.0001** |
| **Diabetes mellitus** | **Yes vs. no** | **1.64** | **1.22-2.22** | **0.0012** |
| **History of CVD** | **Yes vs. no** | **2.17** | **1.57-2.99** | **<0.0001** |
| **Cerebrovascular disease** | **Yes vs. no** | **1.81** | **1.24-2.65** | **0.0019** |
| **PVD** | **Yes vs. no** | **1.66** | **1.18-2.35** | **0.0038** |
| **Albumin (g/L)** | **-5 g/L** | **1.50** | **1.29-1.74** | **<0.0001** |
| Number of antihypertensives | +1 | 0.94 | 0.84-1.06 | 0.3119 |

**Bold** indicates variables that are statistically significant (P<0.05).

CV-MACE, cardiovascular major adverse cardiovascular event; CVD, cardiovascular disease; eGFR, estimated glomerular filtration rate; PVD, peripheral vascular disease.

Table S12. Time to first CV-MACE: Final joint model, sensitivity analysis using Akaike’s information criterion for clinically important variables

| **Dependent variable** | **Change modelled** | **Hazard ratio** | **95% CI** | **Wald P value** |
| --- | --- | --- | --- | --- |
| Estimated eGFR at time t | -5 mL/min/1.73 m^2^ | 1.05 | 0.99-1.11 | 0.0694 |
| Estimated eGFR slope | -5 mL/min/1.73 m^2^ per year | 1.25 | 0.81-1.94 | 0.3080 |
| **Age, years** | **+10 years** | **1.40** | **1.22-1.61** | **<0.0001** |
| **Diabetes mellitus** | **Yes vs. no** | **1.68** | **1.24-2.29** | **0.0008** |
| **History of CVD** | **Yes vs. no** | **2.15** | **1.56-2.97** | **<0.0001** |
| **Cerebrovascular disease** | **Yes vs. no** | **1.81** | **1.24-2.63** | **0.0020** |
| **PVD** | **Yes vs. no** | **1.67** | **1.18-2.36** | **0.0037** |
| Hemoglobin (g/L) | +10 g/L | 1.06 | 0.96-1.16 | 0.2606 |
| **Albumin (g/L)** | **-5 g/L** | **1.53** | **1.30-1.79** | **<0.0001** |
| Number of antihypertensives | +1 | 0.95 | 0.85-1.06 | 0.3336 |

**Bold** indicates variables that are statistically significant (P<0.05).

CV-MACE, cardiovascular major adverse cardiovascular event; CVD, cardiovascular disease; eGFR, estimated glomerular filtration rate; PVD, peripheral vascular disease.

## Supplemental Figures

Fig. S1. Data collection. CV, cardiovascular


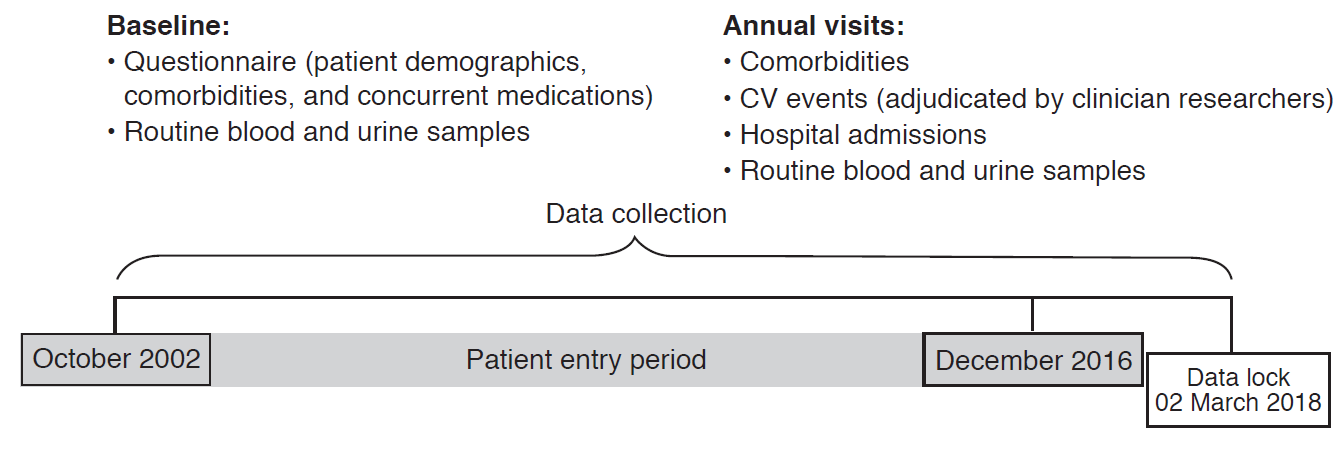


Fig. S2. Patient selection. CKD, chronic kidney disease; eGFR, estimated glomerular filtration rate; NDD, non–dialysis-dependent; SKS, Salford Kidney Study


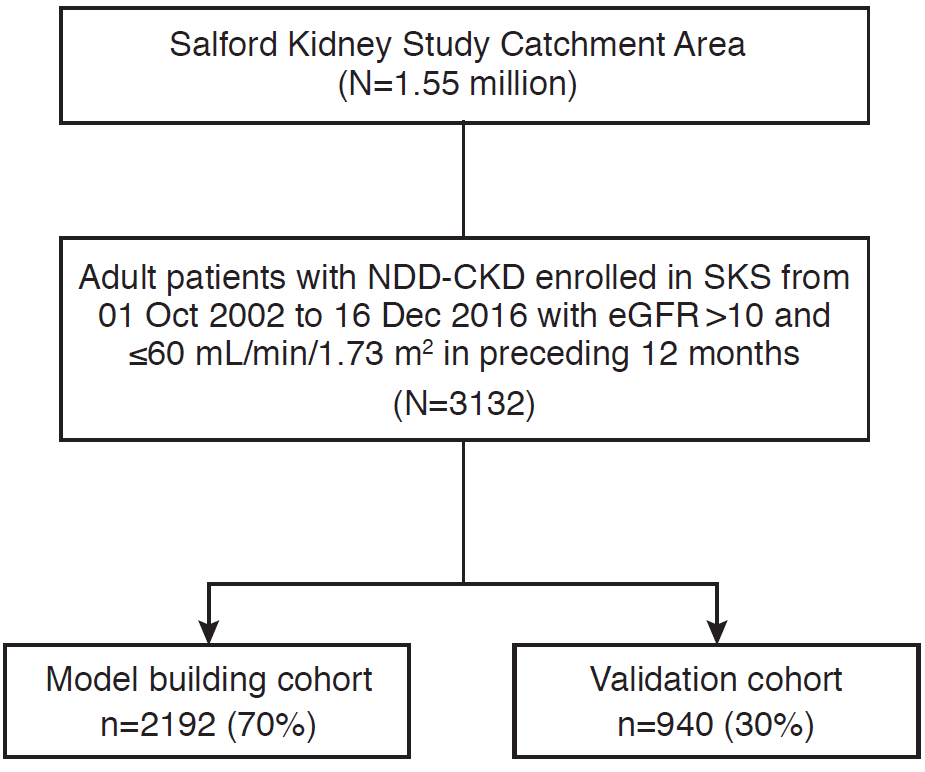


Fig. S3. Kaplan-Meier survival estimate of myocardial infarction in patients with non‒dialysis-dependent chronic kidney disease by chronic kidney disease stage


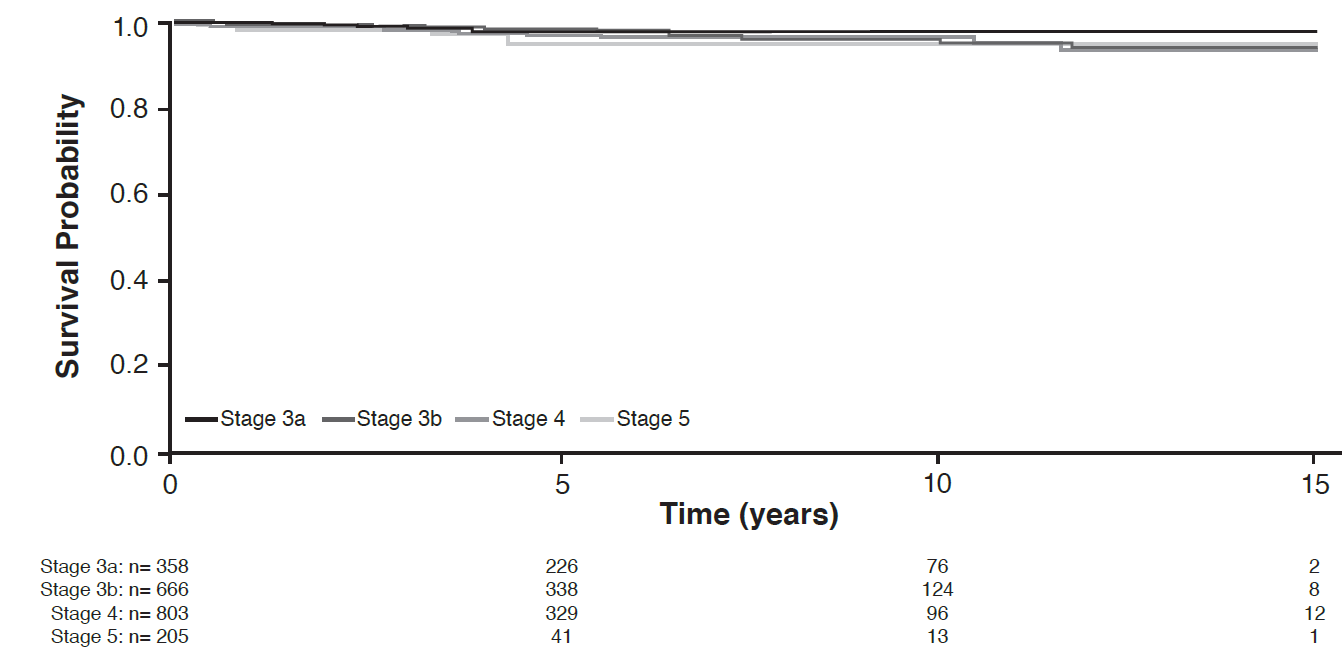


Fig. S4. Kaplan-Meier survival estimate of renal replacement therapy in patients with non‒dialysis-dependent chronic kidney disease by chronic kidney disease stage


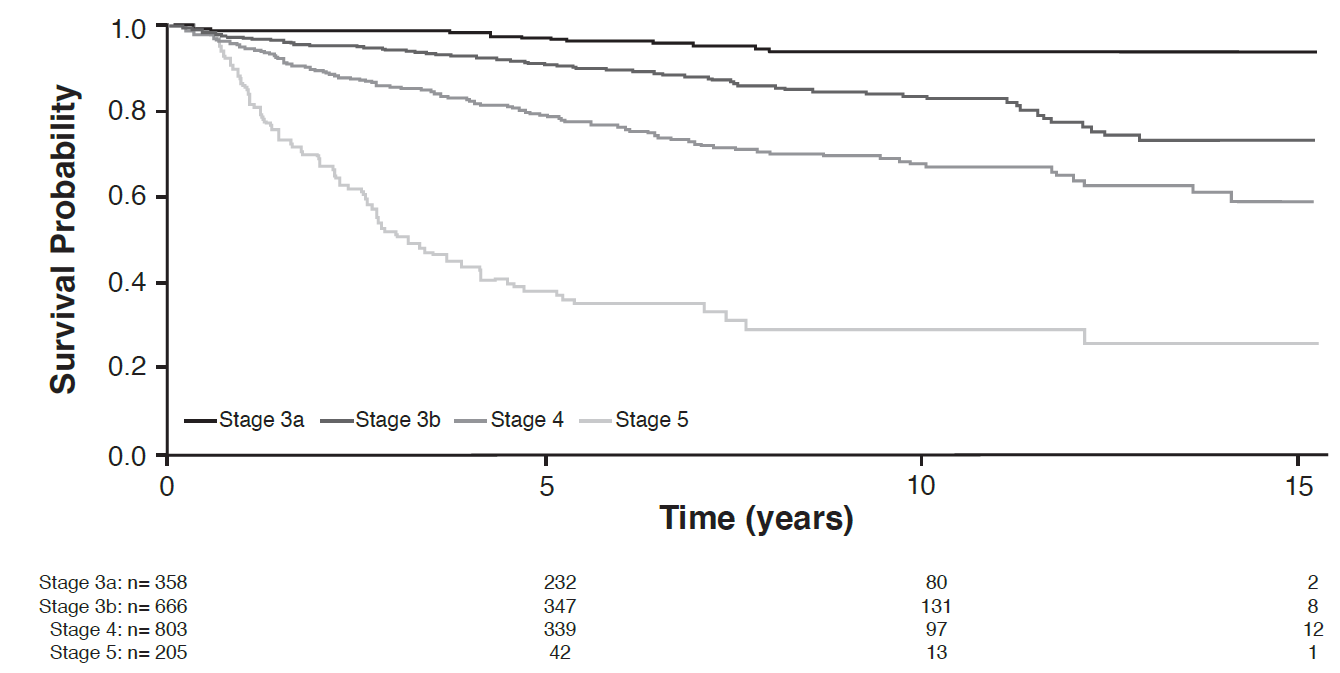


Fig. S5. Post hoc analysis: Kaplan-Meier survival estimate of MACE (composite of non-fatal myocardial infarction, non-fatal stroke, or all-cause mortality) in patients with non‒dialysis- dependent chronic kidney disease by chronic kidney disease stage. MACE, major adverse cardiovascular event


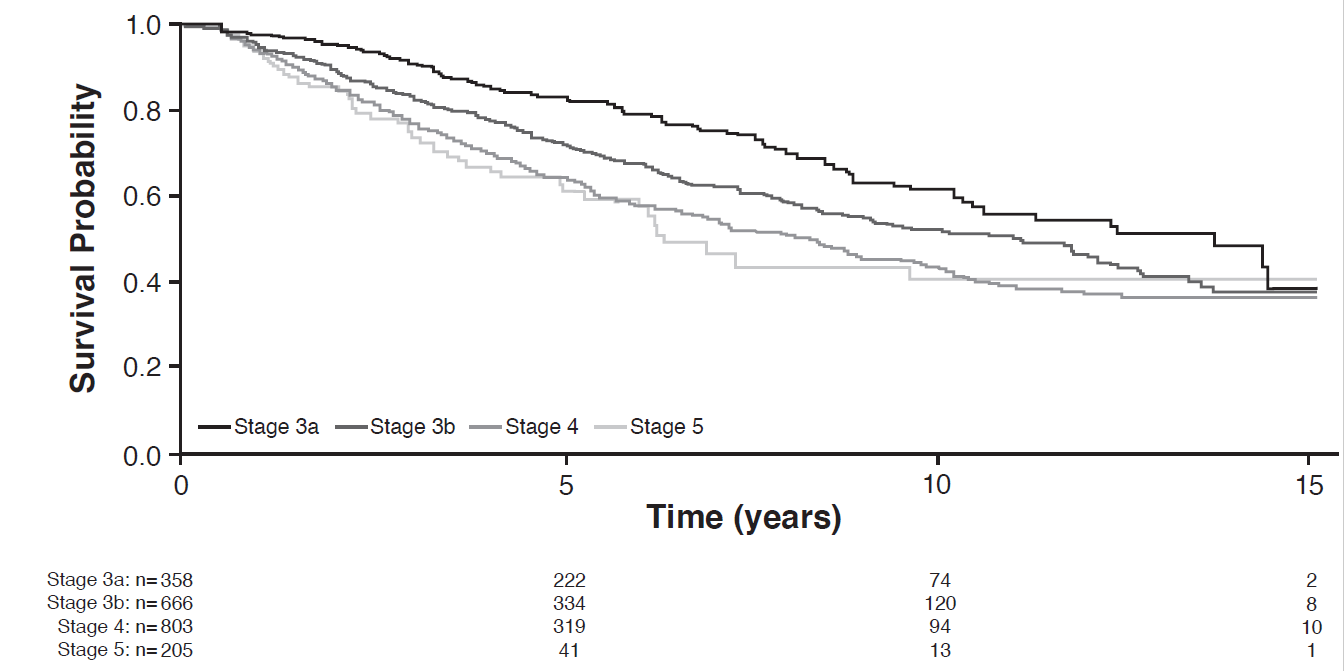


**Fig. S6.** Time to first major adverse cardiovascular event (composite of non-fatal myocardial infarction, non-fatal stroke, and/or cardiovascular mortality) in patients with non‒dialysis- dependent chronic kidney disease: ROC curves for the joint model prediction of cumulative risk at (a) 3 years, (b) 5 years, and (c) 10 years. Only results for the first multiple imputation dataset are shown. Per Safari et al [1], AUC = 1: perfect model, 0.8-0.9: good, 0.7-0.8: fair, <0.7: not considered accurate. AUC, area under the curve; ROC, receiver operating characteristic


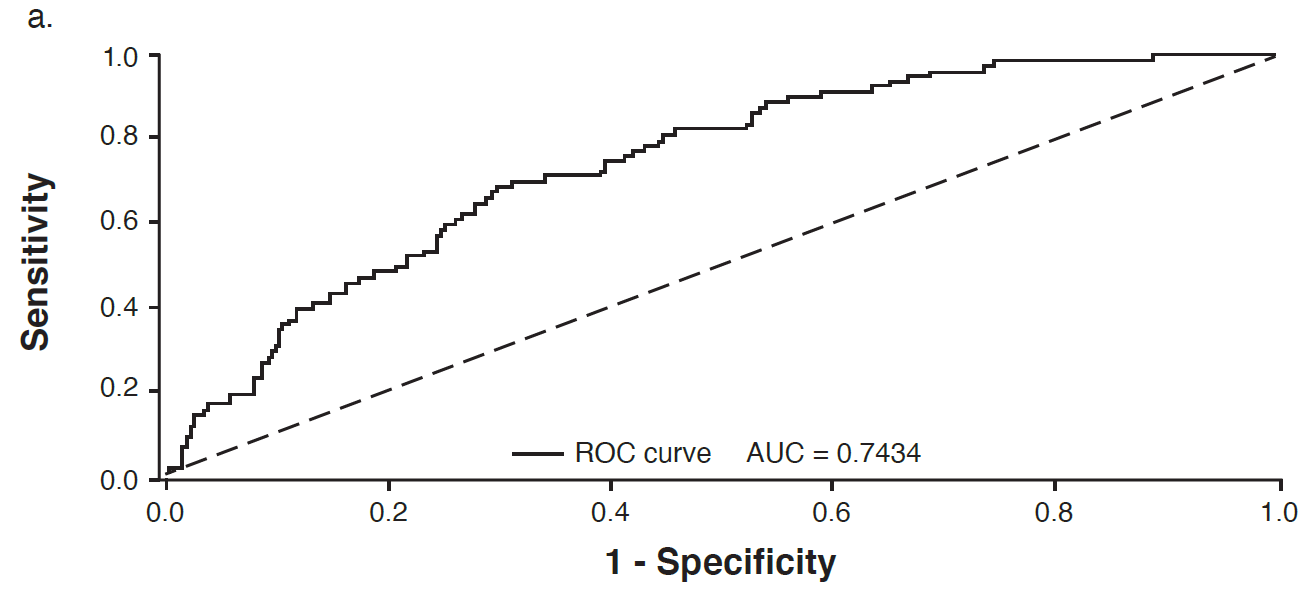


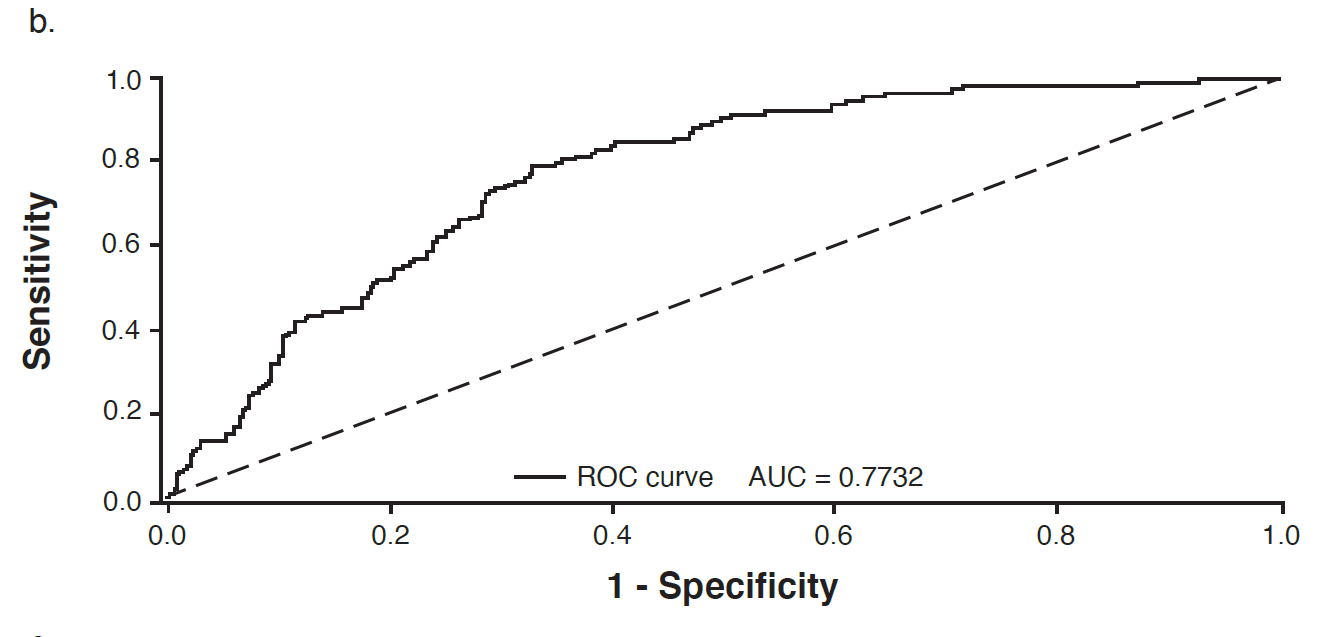


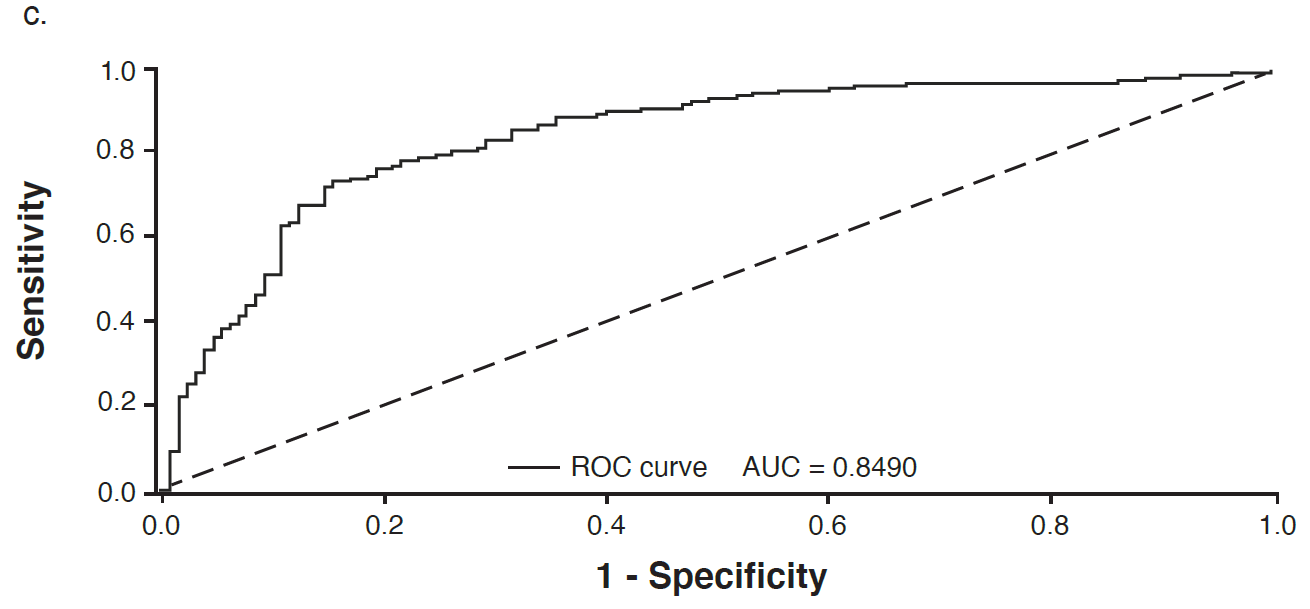


1. Safari S, Baratloo A, Elfil M, Negida A (2016) Evidence based emergency medicine; part 5 receiver operating curve and area under the curve. Emerg (Tehran) 4:111-113.
